# Slc1a3-2A-CreERT2 mice reveal unique features of Bergmann glia and augment a growing collection of Cre drivers and effectors in the 129S4 genetic background

**DOI:** 10.1038/s41598-021-84887-2

**Published:** 2021-03-08

**Authors:** Lech Kaczmarczyk, Nicole Reichenbach, Nelli Blank, Maria Jonson, Lars Dittrich, Gabor C. Petzold, Walker S. Jackson

**Affiliations:** 1 Department of Biomedical and Clinical Sciences, Wallenberg Center for Molecular Medicine, Linköping University, Linköping, Sweden; 2 German Center for Neurodegenerative Diseases (DZNE), Bonn, Germany; 3 Division of Vascular Neurology, University Hospital Bonn, Bonn, Germany

**Keywords:** Neuroscience, Glial biology, Astrocyte

## Abstract

Genetic variation is a primary determinant of phenotypic diversity. In laboratory mice, genetic variation can be a serious experimental confounder, and thus minimized through inbreeding. However, generalizations of results obtained with inbred strains must be made with caution, especially when working with complex phenotypes and disease models. Here we compared behavioral characteristics of C57Bl/6—the strain most widely used in biomedical research—with those of 129S4. In contrast to 129S4, C57Bl/6 demonstrated high within-strain and intra-litter behavioral hyperactivity. Although high consistency would be advantageous, the majority of disease models and transgenic tools are in C57Bl/6. We recently established six Cre driver lines and two Cre effector lines in 129S4. To augment this collection, we genetically engineered a Cre line to study astrocytes in 129S4. It was validated with two Cre effector lines: calcium indicator gCaMP5g-tdTomato and RiboTag—a tool widely used to study cell type-specific translatomes. These reporters are in different genomic loci, and in both the Cre was functional and astrocyte-specific. We found that calcium signals lasted longer and had a higher amplitude in cortical compared to hippocampal astrocytes, genes linked to a single neurodegenerative disease have highly divergent expression patterns, and that ribosome proteins are non-uniformly expressed across brain regions and cell types.

## Introduction

Humans are highly diversified on multiple levels which influences vulnerability to diseases and responsiveness to medical treatments. Phenotypic differences are reflected in gene- and protein expression profiles and similar variations are observed in other mammals, including laboratory mice^1^. Genetic background is a primary factor affecting disease phenotypes in mouse models^2–4^ and a notorious experimental confounder. Therefore, inbred mouse strains, in which all individuals are genetically identical, are often employed to limit phenotypic variation. C57Bl/6 includes numerous sub-strains, collectively known as Black 6 or B6, and is the most commonly used inbred mouse strain with nearly 46,000 publications on Pubmed as of July 2020^5,6^. Their popularity stems from some important features. First, since they carry a recessive mutation resulting in black fur, they were widely used during the early days of gene-targeting experiments as sources of host blastocysts and breeding partners for chimeras. This provided an invaluable screening tool for quick exclusion of non-chimeric mice and progeny of chimeric mice that did not inherit genetically engineered ES cell genome, simply by inspecting fur colors, a convenience still widely used today. Second, B6 mice are more susceptible than most other strains to certain drugs and self-administer them when given the chance^7–9^. They also perform well in learning and memory paradigms, as they are inclined to explore more than other strains^10–12^. Finally, B6 mice are very active, which is a useful characteristic for studying metabolic rates and effects of exercise on the body^13^.

Congruent with these characteristics, using automated mouse behavior analysis (AMBA) we observed hyperactivity, especially at night^14,15^, in a subset of mice congenic for a B6 background (hereafter, for the introduction and results sections, B6 refers specifically to C57Bl/6N). Since the hyperactivity was observed in knock-in controls, we suspected it was due to the genetic background rather than the engineered mutations. This was alarming since nocturnal hyperactivity in only a subset of mice would result in those individuals being more tired during the day, which would likely go unnoticed. Indeed, sleep quality affects gene expression at genome and transcriptome levels^16^ and poor sleep quality is linked to diseases, including neurodegeneration, cancer, and diabetes^17–20^. We also observed an obese subset which was troublesome since obesity also leads to gene expression differences in the brain^21^. A subset of individuals of an inbred strain with atypical characteristics makes the population heterogeneous, diminishing the value of using an inbred strain. We therefore tested an alternative genetic background, 129S4 (formerly 129 Sv/Jae, hereafter S4). This strain is used in amphetamine research^22,23^ and a quantitative electroencephalography (EEG) study revealed several differences in sleep physiology between S4 and B6^24^. Hours long AMBA experiments with S4 mice^22^ suggested they were calmer than B6 mice leading us to suspect they would also be calmer through an entire 24-h period. Consequently, the differences between mouse strains in this domain might translate to strain-dependent experimental differences in mouse models of disease, highlighting the importance of choosing the optimal genetic background that either enables, or at least does not interfere with, the goals of the experiment.

Here, using AMBA, we systematically compared the home cage behavior of S4 and B6 mice and verified the pronounced intra-strain and within-litter variabilities in B6. Concerned these differences could mask subtle gene expression characteristics of mild phases of neurodegenerative and psychiatric disorders we have backcrossed multiple Cre and Cre reporter lines to S4^25^, which was very time consuming. Unfortunately, the connexin-43 astrocyte-specific Cre line we used to study stroke^26^ was impractical because they could not be maintained as homozygotes^27^ and breeding heterozygotes provided few age and sex matched disease and control animals. To meet the need for a better Cre line for astrocytes while avoiding extensive backcrossing of existing lines we generated an isogenic S4 inducible Cre driver line. These mice express CreERT2^28^ from the solute carrier family 1 member 3 (Slc1a3) locus^29^ while preserving expression of the endogenous protein using the 2A peptide bicistronic expression method^30^. The ability of *Slc1a3* gene elements to drive high and specific transgene expression in astrocytes has been previously demonstrated^31–34^, but a knock-in approach to express a transgene from the endogenous *Slc1a3* locus while preserving expression of the endogenous protein has not been reported. Here we attempted to fill this void. We demonstrate the functionality of the Slc1a3-2A-CreERT2 mouse line with two separate Cre reporter lines. The GCaMPg5 calcium sensor revealed functional differences between hippocampal and cortical astrocytes in vivo. RiboTag mice were used to compare translating mRNAs from cerebellar astrocytes, almost exclusively comprising Bergmann glia, with astrocytes of the rest of the brain, providing insights into the translatome of this unique cell type.

## Results

### B6 mice are variably hyperactive at night

**Figure 1:**
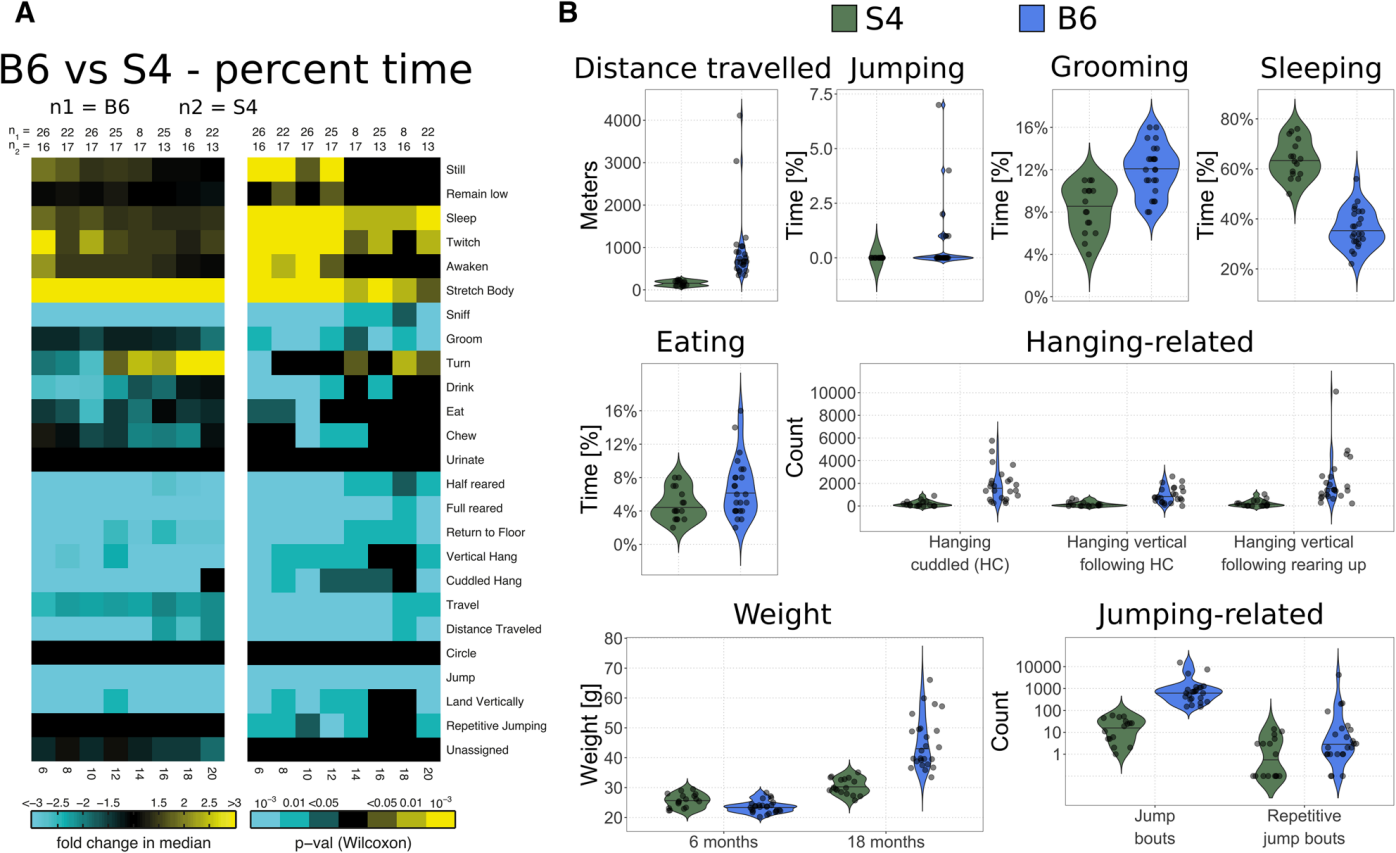
Home cage automated behavioral analysis. (**A**) Heat map showing statistical summary of longitudinal video monitoring analysis performed at 6, 8, 10, 12, 14, 16, 18, and 20 months of age on female mice. The number of mice at each timepoint is represented on the top of the heatmap as n1 for B6 and n2 for S4. Yellow tiles represent features present more in S4 than B6, whereas cyan tiles represent the opposite. The behavior “urinate” was not reliably scored so was disabled in this analysis. Otherwise, the heatmap displays all metrics studied. Two-tailed Wilcoxon rank sum (non-parametric) test. A p-value < 0.05 was considered significant. Multiple testing corrections were deemed unnecessary since nearly all metrics showed large differences. (**B**) Violin plots for select behaviors represent multiple metrics where B6 data where very spread out (tall and narrow violins) and the population is variable. In contrast, the violins in S4 are short and wide, indicating the population is very similar. The weight depicts data for 6 and 18 months, all other charts represent data at 6 months. Note the scale is log_10_ for the “Jumping-related” panel. While both B6 and S4 demonstrate a wide range of jumping, the overall range in B6 is 100-fold higher than S4.

To compare behavioral characteristics of B6 and S4 strains, we performed AMBA using videos of mice in standard cages recorded for 24-h periods every 2 months starting at 6 months of age. The videos were scored automatically by computer software as described previously^14,35^. A comparison across ages revealed many differences in activities of adult S4 and B6 mice, even into old age (Fig. 1A). Importantly, a closer inspection of data from individual mice revealed a subset of B6 mice demonstrating hyper-repetitive activities for behaviors requiring exertion (e.g., jumping, walking, rearing, hanging, etc.) or feeding (Fig. 1B). Moreover, individuals hyperactive in one behavior were often not hyperactive in others. For example, in 24 h, one mouse traveled 4,000 m whereas another jumped for over 3 h and yet another kept its snout in the food bin “eating” for 3.8 h (Supplementary Fig. S1). Interestingly, another subset of B6 became much fatter than the rest (Fig. 1B). For example, at 18 months 5 of 25 B6 mice were over 20% heavier than the median weight (42.4 g), 4 of which were over 30% heavier. The fat B6 mice were not the same as those with the highest eating scores (Supplementary Fig. S1), indicating the high eating scores included activities besides consumption of food, possibly excessive gnawing. Remarkably, much of the hyper-repetitive behaviors occurred during the dark cycle, when mice are typically not observed by human investigators (Supplementary Movie S1, https://tinyurl.com/wcmz9zf). In contrast, S4 mice demonstrated no hyper-repetitive behaviors and 0 of 17 S4 mice became more than 20% heavier than the median weight (29.9 g) despite eating the same standard chow as the B6 mice. Such drastically different behaviors and weights likely have an impact on metabolism and physiology, and finally on gene expression^36^. Therefore, to reduce the inter-replicate variability in gene expression studies, we have begun to use the S4 genetic background in most of our experiments.

### Generation of the S4 Slc1a3-2A-CreERT2 mouse line

**Figure 2:**
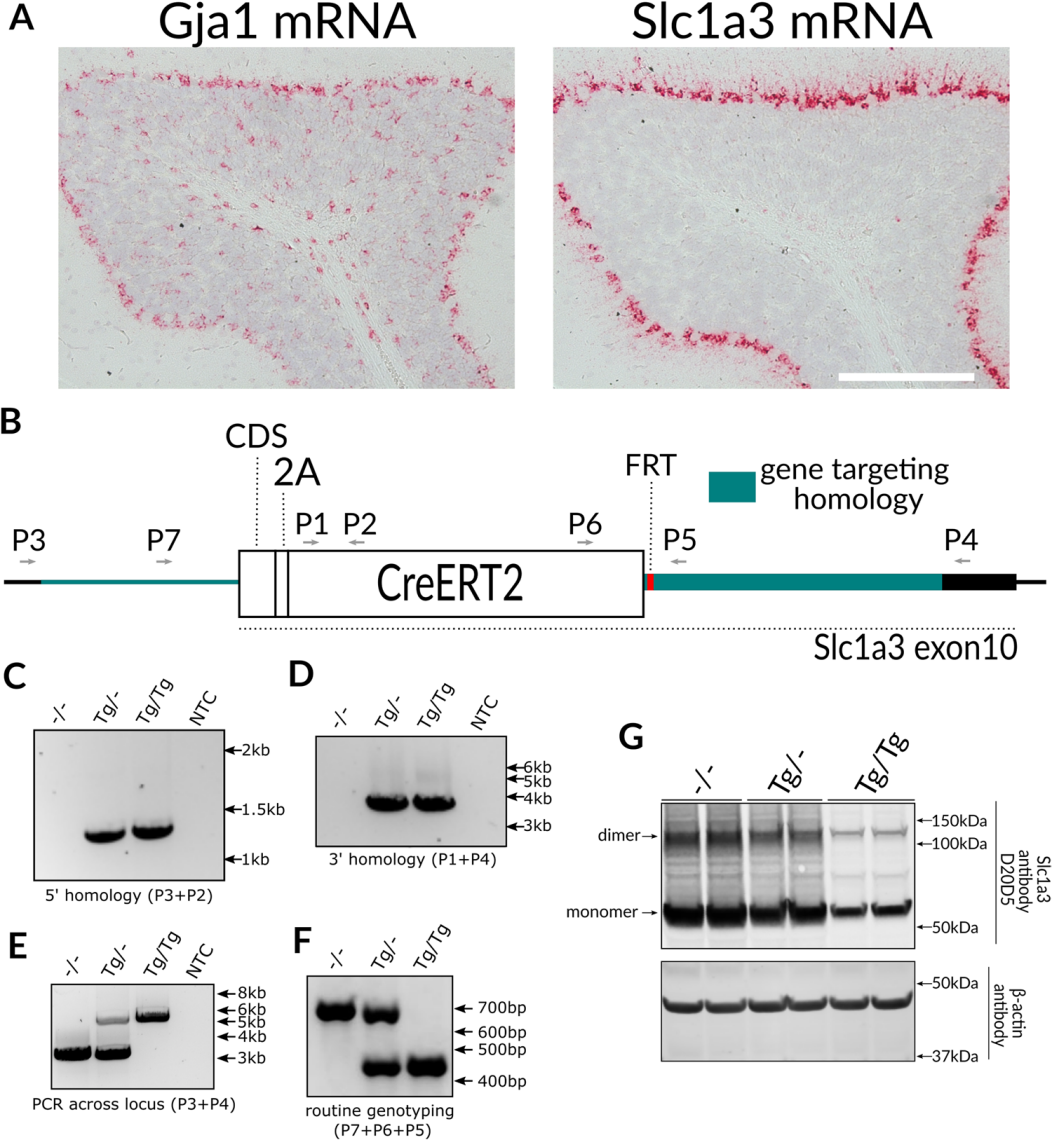
Generation of Slc1a3-2A-CreERT2 mice in 129S4 background. (**A**) In situ hybridization for Gja1 and Slc1a3 of a 10 months old mouse brain. Note disparate expression pattern in the cerebellum, where Slc1a3 is expressed almost exclusively in Bergman glia. **(B)** Schematic of Slc1a3-2A-CreERT2 targeted locus (elements are to scale). (**C**–**F**) Genotyping PCR. PCRs with primers flanking 5; homology region (**C**), 3′ homology region (**D**), flanking the whole targeted locus (we never managed to obtain the targeted band for heterozygote, probably due to WT target preference of the assay), (**E**), and routine genotyping assay (**F**) were shown. (**G**) Immunoblot using an anti-Slc1a3 antibody showing Slc1a3 protein levels in WT, heterozygous and homologous knock-in mice. PCR oligo sequences are in Supplementary Table S1 and uncropped images used to prepare panels C-G are shown in Supplementary Fig. S2.

During brain development, neural precursor cells (NPCs) express many genes typically considered markers for astrocytes^37,38^. Therefore, constitutive expression of Cre with astrocyte specific marker genes or promoter elements results in recombination in cells that later become neurons^39,40^. To avoid this ectopic activation, we used an inducible Cre variant, CreERT2, that can be chemically activated after development to circumvent the problem of neuronal targeting^28,32^. We chose to use a knock-in approach since bacterial artificial chromosome (BAC) transgenes can be highly disruptive to the genome, both through the insertion of a very large transgene and massive deletions at the integration site^41,42^. To identify a native gene to carry the CreERT2 coding sequence we employed mRNA in situ hybridization (RNA ISH) to evaluate the native expression patterns of three genes encoding well known astrocyte marker proteins; Aldh1l1 (Aldehyde dehydrogenase 1 family member l1), Gja1 (Gap junction alpha 1, also known as connexin 43) and Slc1a3 (also known as glutamate aspartate transporter (GLAST) or excitatory amino acid transporter 1 (EAAT1)). While Aldh1l1 was weakly detected (not shown), Gja1 and Slc1a3 demonstrated strong expression throughout the brain, very similar to that presented in the Allen Brain Atlas database (https://mouse.brain-map.org/). Although the overall expression of Gja1 was higher than that of Slc1a3, we saw two important advantages with Slc1a3. First, the cerebellar expression of Gja1 was prominent in the Purkinje cell layer (the location of Bergmann glia, BG), in the granule cell layer and in the deep cerebellar nucleus (Fig. 2A). In contrast, cerebellar expression of Slc1a3 was restricted to BG (Fig. 2A). We thought that having a Cre active in a very homogeneous cell type in an easily accessed brain region would be extremely useful, especially for gene expression studies. Second, the homozygous knock-out state is lethal for Gja1 but not Slc1a3, so that in the event of partial gene inactivation of Slc1a3, the line could be maintained as homozygous and the health impact for heterozygous, experimental mice would be negligible. Although the in situ hybridization was done only on adult tissue sections and the expression of Slc1a3 may differ during development, we expected that the expression pattern we observed in adults could be reproduced with Cre by using an inducible system.

To express CreERT2 from the *Slc1a3* locus while preserving expression of the endogenous gene product, we used the 2A peptide strategy (Fig. 2B). A pair of Cas9 nickases targeted to the proximity of the STOP codon were used to ensure efficient targeting with short (1.1 kb left and 1.6 kb right) homology arms. A positive ES cell selection cassette (FRT-NeoR-FRT, FNF) was placed downstream of CreERT2 in the 3′ untranslated region (UTR). The FNF cassette was later removed by breeding to ROSA26-Flpo deleter mice on the S4 background^43^. To verify gene targeting fidelity, we performed PCR across each homology region (Fig. 2C,D), as well as across the whole locus, using primers located outside of the regions present in the targeting vector (Fig. 2E). Finally, we verified that wild type Slc1a3 was absent in homozygous Slc1a3-2A-CreERT2 mice (Fig. 2F). All this unambiguously verified that no undesirable duplication or O-type recombination took place^44^. Immunoblotting on total brain lysates showed expression of Slc1a3 protein was markedly decreased in heterozygotes, and further decreased in homozygotes (Fig. 2G). Although 2A-driven separation is sometimes incomplete, such a product does not appear to have accumulated in these mice since it would appear as a band absent from wild-type, present in heterozygotes, and more abundant in homozygotes. Importantly, homozygous mice appear and behave normally, and we maintain the line as homozygous. To test for reproductive issues, we compared the litter sizes of all S4 lines that we keep (the lines we bred to, or made directly in, S4 background are listed in Table 1) and observed no differences (Supplementary Fig. S3). In the experiments that follow, homozygous Slc1a3-2A-CreERT2 mice were crossed with Cre reporter strains such that experimental mice were heterozygous for both transgenes.

**Table 1:** Knock-in mouse lines backcrossed to S4.

Line	Feature	% S4^a^	PMID	Reference
PV-IRES-Cre	Marks PV neurons	99.8	15836427	Hippenmeyer, 2005^45^
SST-IRES-Cre	Marks SST neurons	99.8	21943598	Taniguchi, 2011^46^
Gad2-IRES-Cre	Marks GABA neurons	99.8	21943598	Taniguchi, 2011^46^
vGlut2-IRES-Cre	Marks glutamatergic neurons	100	21745644	Vong, 2011^47^
Calb2-IRES-Cre	Marks calbindin2 neurons	91.2^b^	2194398	Taniguchi, 2011^46^
Slc1a3-2A-CreERT2	Marks astrocytes	100^c^	This report	This report
Cx3cr1-CreERT2	Marks brain macrophages	91.0^b^	23273845	Yona, 2013^48^
LSL-Tagger	Tagger (reporter, IP tool)	98.6^b^	31393866	Kaczmarczyk, 2019^25^
Rpl22-HA	RiboTag (reporter, IP tool)	99.8	19666516	Sanz, 2009^49^
HdhQ200	Disease model	100	11152661	Lin, 2001^50^

*PV* parvalbumin, *SST* somatostatin, *GABA* gamma-aminobutyric acid, *IP *immunoprecipitation.

^a^Based on genotyping 347 loci across the genome that discriminate S4 from B6.

^b^These lines were backcrossed four more generations after genotyping analysis.

^c^This line was made directly in S4.

### Calcium levels are highly dynamic in cortical but not hippocampal astrocytes

The CreERT2 fusion protein is engineered to be anchored in the cytoplasm through binding with heat shock protein 90 (Hsp90)^28^. In the presence of 4-hydroxytamoxifen (4-OHT) Hsp90 releases CreERT2 which is then promptly shuttled into the nucleus for recombination. Peripheral application of tamoxifen also works as it is processed to 4-OHT by the liver. To test the functionality of our Slc1a3-2A-CreERT2 line we bred them to a Cre reporter line, PC::G5-tdT, that encodes a green fluorescent protein fused to the calcium binding protein calmodulin (GCaMP5), useful for measuring changes to intracellular calcium levels^51^. This transgene also encodes a separate red fluorescent protein, tdTomato, that provides stable fluorescence, independent of calcium fluctuations, thereby serving as a baseline reference for fluorescence emission. This line was chosen as it is a very popular tool for in vivo astrocyte studies. We activated expression with tamoxifen in three months old mice.

**Figure 3:**
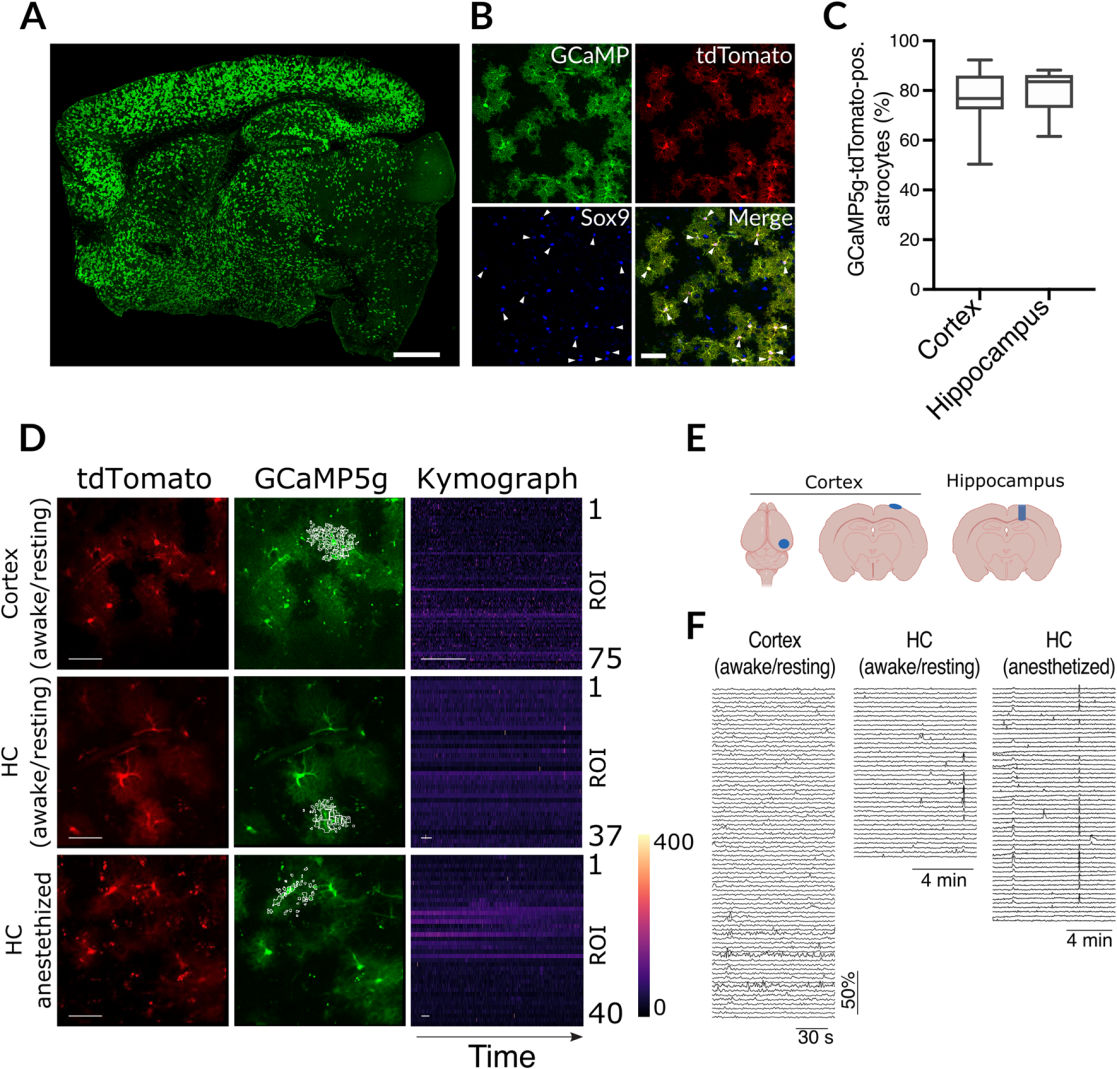
Calcium imaging. (**A**,**B**) Fluorescent imaging of Slc1a3-CreERT2::GCaMP5g-tdTomato-loxP using antibodies against GFP, the astroglial marker Sox9, and the native fluorescence of tdTomato, showed widespread and strong Cre recombination specifically in astrocytes (arrowheads; scale bars, 1 mm and 50 µm, respectively; Sox9 nuclear marker was used to identify astrocytes. (**C**) Quantification of recombination efficacy in hippocampus and cortex; reporter expression was normalized to all Sox9-positive cells. (**D**) Representative examples of calcium activity under awake/resting conditions in the cortex, and under awake/resting conditions or anesthesia in the hippocampus (HC). No changes were seen in tdTomato native fluorescence (scale bars, 50 µm), indicating stable imaging conditions over time, while activity-evoked calcium changes could be detected using GCaMP5g native fluorescence. The kymographs represent color-coded calcium changes over time (scale bars, 30 s) in distinct regions of interest (ROI) from individual astrocytes (marked by white boxes in the GCaMP5g images). (**E**) Locations of cranial windows implanted over cortex and hippocampus. (**F**) Individual traces for all conditions and regions. The traces correspond to ROIs and kymographs depicted in D. Mice were 4 months of age.

We first determined if activation was specific to astrocytes by labeling formaldehyde fixed brain sections with anti-GFP antibodies specific to the reporter and the astrocyte-specific nuclear marker Sox9^52^. These experiments revealed widespread and specific Cre recombination in about 80% of all Sox9-positive hippocampal and cortical astrocytes (Fig. 3A–C). Astrocytes communicate within the astroglial network and with other cell types mainly through intracellular calcium elevations, important in health and disease^53–55^. Hence, to estimate the usefulness of this Cre line for functional astroglial imaging, we imaged GCaMP5 changes through cortical windows in the cortex and hippocampus under awake and resting conditions as well as under anesthesia (Fig. 3D–E) using two-photon microscopy three weeks after Cre induction. These experiments showed that calcium signals in the cortex in awake mice under resting conditions had a higher amplitude with a longer duration (FDHM) than in the hippocampus of awake and resting mice or in the hippocampus of anesthetized mice (Fig. 3F).

### Bergmann glia express markers similar to cerebral astrocytes, but their translatomes are profoundly different

**Figure 4:**
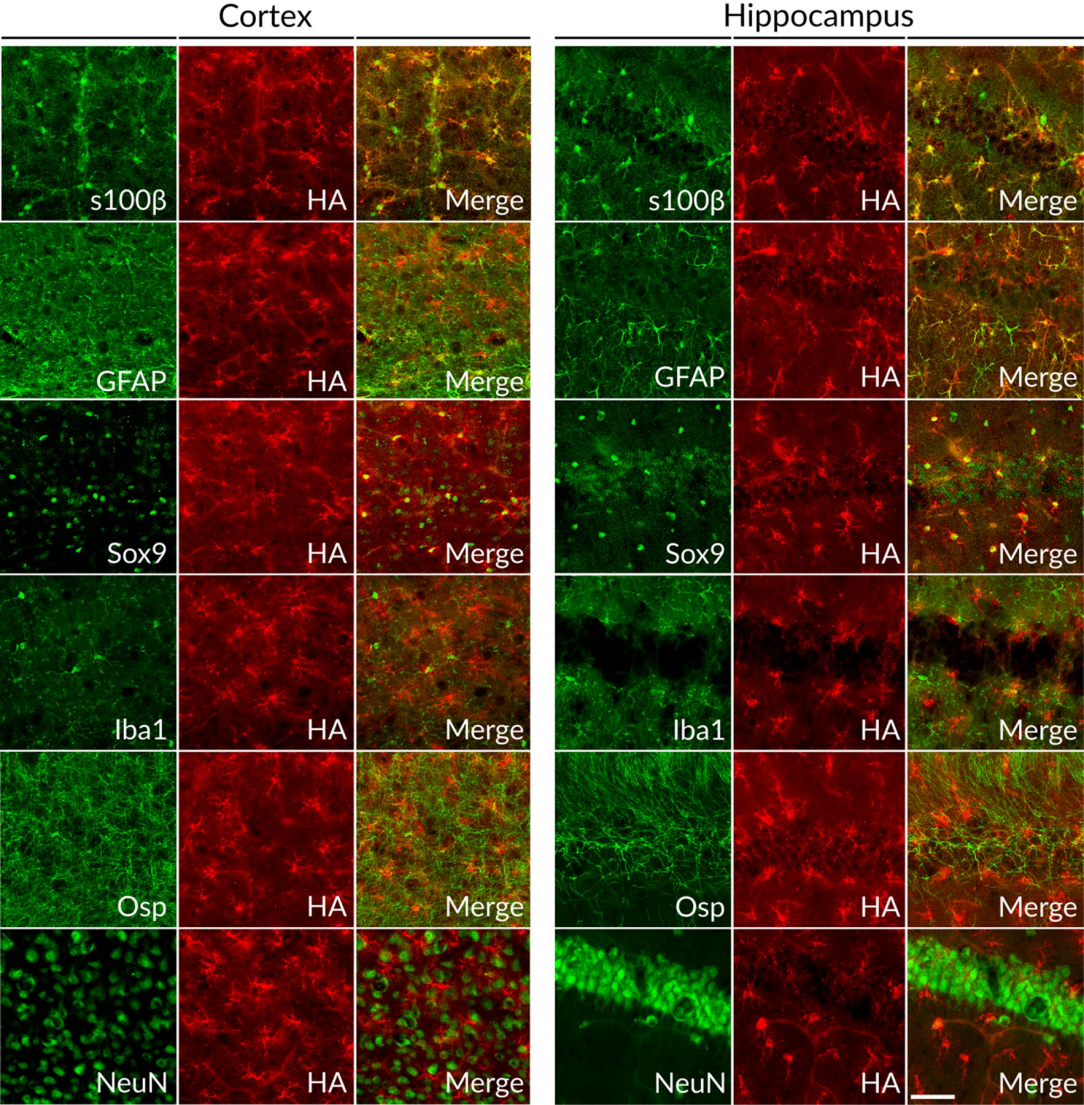
Immunofluorescence staining of Slc1a3-CreERT2::RiboTag mice. Representative images from hippocampus and cortex of 7 weeks old mice. RiboTag is a Cre reporter line that upon recombination leads to the expression of an HA (hemagglutinin)-tagged ribosome protein. For each marker, co-staining with anti-HA antibody (HA; RiboTag, red) was used to mark recombined cells; *S100β* calcium binding protein (astroglial marker), *GFAP* glial fibrillary acidic protein (astroglial marker), *Sox9* SRY-Box Transcription Factor 9 (astroglial marker), *Iba1* ionized calcium binding adaptor molecule (microglial marker), *Osp* oligodendrocyte surface protein, *NeuN* neuronal nuclei. Scalebar = 50 μm.

We next sought to characterize the diversity of mRNAs undergoing translation (translatome) in Bergmann glia of the cerebellum and astrocytes of the rest of the brain (hereafter BG and CA, respectively). To this end we crossed Slc1a3-2A-CreERT2 and RiboTag mice^49^ congenic for S4^26^. RiboTag mice carry a floxed allele of the endogenous Rpl22 (large subunit ribosomal protein 22) gene that expresses an HA (hemagglutinin) antibody epitope-tagged version of Rpl22 following Cre recombination. CreERT2 was induced with daily intraperitoneal tamoxifen injections for three consecutive days and mice were sacrificed 4 days after the final injection. To evaluate the specificity of recombination, we co-labeled formaldehyde fixed brain sections with an HA antibody for detection of RiboTag and antibodies against marker proteins specific for astrocytes or non-astrocyte cell types. HA positive cells looked like astrocytes and expressed astrocyte marker proteins such as S100ß, Sox9 or GFAP (glial fibrillary acidic protein), but not Osp (oligodendrocyte-specific protein), NeuN (neuronal nuclei), or Iba1 (ionized calcium-binding adaptor molecule 1, microglial marker) (Fig. 4). Thus, as observed with GCaMP5, Slc1a3-2A-CreERT2 activated RiboTag expression in the majority of astrocytes, and only astrocytes.

**Figure 5:**
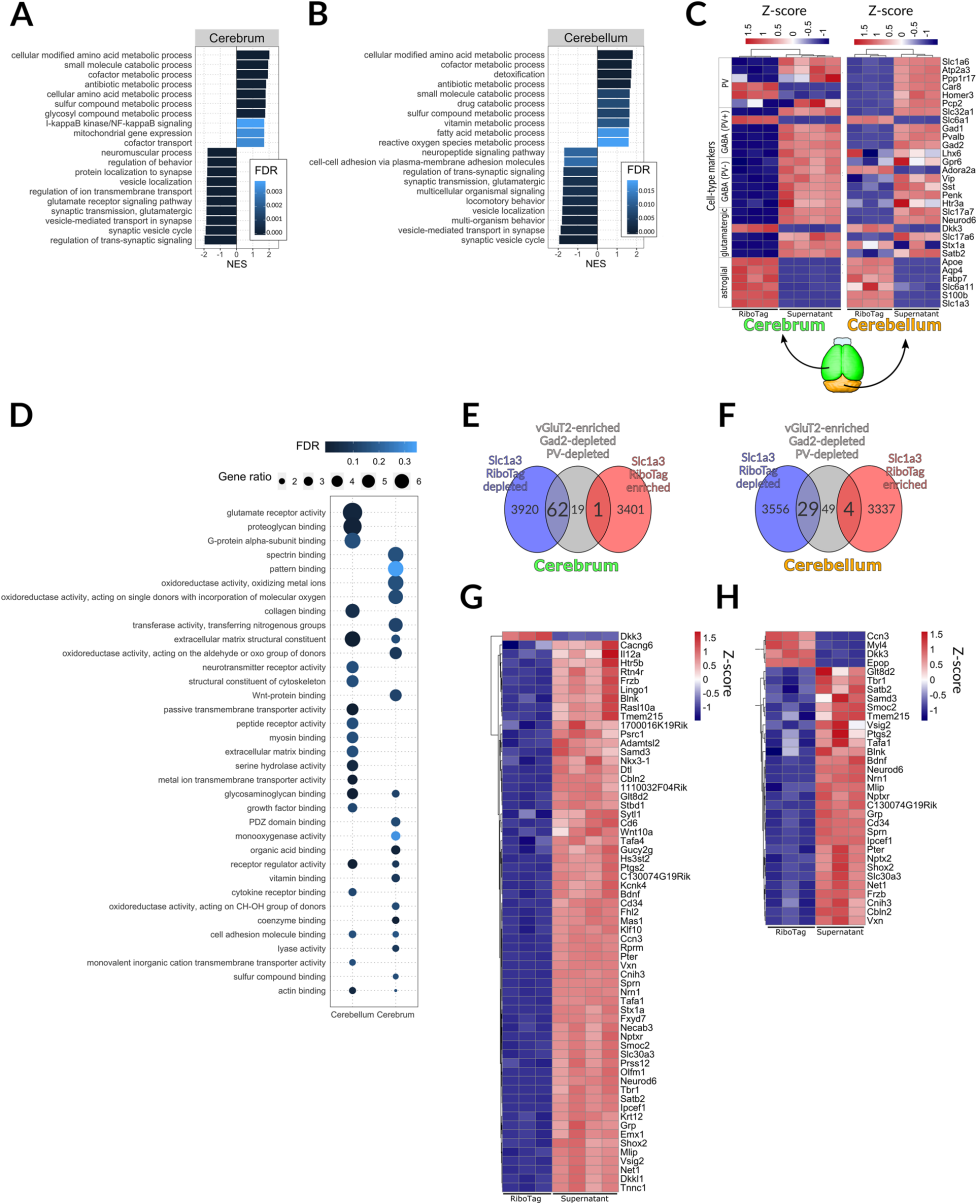
Assessment of RiboTag analysis specificity. (**A**,**B**) Gene set enrichment analysis (GSEA) of RiboTag IP in cerebrum (**A**) and cerebellum (**B**); negatively and positively enriched genes correspond to gene sets functionally related to neuronal and glial function, respectively; analysis was done using WebGestalt^105^ using molecular function GO terms, genes were ranked as described in the methods section and top 10 hits for each region were shown; NES = normalized enrichment score. (**C**) Heatmap showing relative distribution of RiboTag RPKM values for genes for cell type-enriched mRNAs that we previously used to validate cell type specificity of gene expression in Tagger mice^25^. Each column represents one biological replicate. Z-score was calculated as follows: ${\mathrm{Z }}= ({\mathrm{x }}-{\mathrm{ meanrow}}\left({\mathrm{x}}\right))/{\mathrm{SD}}({\mathrm{row}})$, where SD is standard deviation. (**D**) GO analysis of genes determined as enriched (FDR < 0.05, LFC > 1) in BG and AC; top 20 GO categories (molecular function, MF) for each cell population were shown. (**E**,**F**) Venn diagrams showing overlaps of significantly enriched (red circle, LFC > 1, FDR < 0.05) and depleted (blue circle, LFC < − 1, FDR < 0.05) genes in Slc1a3-2A-CreERT2::RiboTag with genes enriched in glutamatergic neurons in vGluT2-Cre::Tagger mice. Grey circle contains genes that are enriched in vGluT2 + cells (LFC > 1, FDR < 0.05), and simultaneously not enriched in PV + neurons and Gad + neurons (LFC < 0, FDR < 0.05 for both cell types). Note that in case of our Tagger analysis whole brain was used, hence the expected overlap (circle intersections; genes enriched in vGluT2 should be depleted in astroglia and vice versa) is more pronounced for the cerebrum (more similar to Tagger samples) (**E**) than for the cerebellum (**F**). (**G**,**H**) Heatmaps showing genes from the intersections of the Venn diagrams in E and F.

We next studied the translatome of BG and CA by capturing HA-tagged ribosomes with immunoprecipitation (IP) and analyzing the captured mRNAs with next generation sequencing (NGS). We first performed a principal component analysis in which the first principal component separated both RiboTag IPs from their corresponding total RNA samples in the same direction and to a similar extent, while the second principal component separated the samples based on the brain region to the same extent for input and IP, indicating that the translatomes of BG and CA are very different (Supplementary Fig. S4). We then tested if the differentially expressed genes were sufficient to reveal the identity of the cells from which the mRNAs were captured. Gene set enrichment analysis (GSEA) demonstrated that the top 10 pathways enriched in IPs from both regions involved processes of metabolism, catabolism, and detoxification, known core functions of astrocytes (Fig. 5A,B). In contrast, the top 10 pathways depleted from IPs from both regions involved processes related to neuronal activities and synaptic structures (Fig. 5A,B). These results indicate that an unbiased analysis was sufficient to establish that the IP-captured samples were enriched for mRNAs expressed by astrocytes and depleted for mRNAs expressed by neurons.

We further validated the cell type specificity of the captured mRNAs by analyzing a list of marker genes generated with RiboTag data^25^. As expected, markers specific for astrocytes (Apoe, Aqp4, Fabp7, Slc6a11, S100b and Slc1a3) were highly enriched in RiboTag IP samples, whereas markers for a wide diversity of neurons were depleted (Fig. 5C), with some notable exceptions. Adora2a (adenosine receptor 2a) is commonly considered a marker for astrocytes but is also a marker for striatal GABAergic neurons. Consistent with this, Adora2a was enriched in BG RiboTag samples but depleted from CA RiboTag fractions, where the striatum is located (Fig. 5C). Slc1a6 is a GABA transporter and was included in our list as a marker of GABAergic neurons. However, in the current study it appeared to be preferentially expressed by BG and CA, suggesting a more prominent role for GABA uptake by Slc1a6 for astrocytes than neurons. Likewise, Gria1 and Gria4, both involved in AMPA signaling in Bergmann glia^56,57^, were enriched in RiboTag samples from BG but not CA. We then compared these data to a larger manually curated list based on literature with the same overall result that astroglial markers were enriched and markers of other cell types were depleted (Supplementary Fig. S5). We then did gene ontology (GO) analysis on genes significantly different (LFC > 1, FDR < 0.05) between BG and CA. Consistent with previous observations of expression of glutamate receptors by BG, the most enriched molecular function GO category in BG was “glutamate receptor signaling” (Fig. 5D). That result motivated us to ask how similar the translatomes of BG are to those of glutamatergic neurons. Employing previous data^25^, we curated a list of mRNAs that are specifically enriched in glutamatergic neurons compared to GABAergic and parvalbumin neurons and determined if this selection overlapped with mRNAs that were either enriched or depleted in astrocyte translatomes. We found 82 genes were specific for glutamatergic neurons, of which only 1 was enriched in CA whereas 4 were enriched in BG (Fig. 5E–H). In contrast, 62 and 29 genes were depleted from CA and BG translatomes, respectively (Fig. 5E–H), indicating that glutamatergic-related signaling systems are expressed more in BG, and may be a functional characteristic^58^. Overall, these analyses supported the immunofluorescence study indicating the Slc1a3-2A-CreERT2 line specifically activated RiboTag in astrocytes. Furthermore, they highlighted that the translatomes of BG and CA are remarkably different.

**Figure 6:**
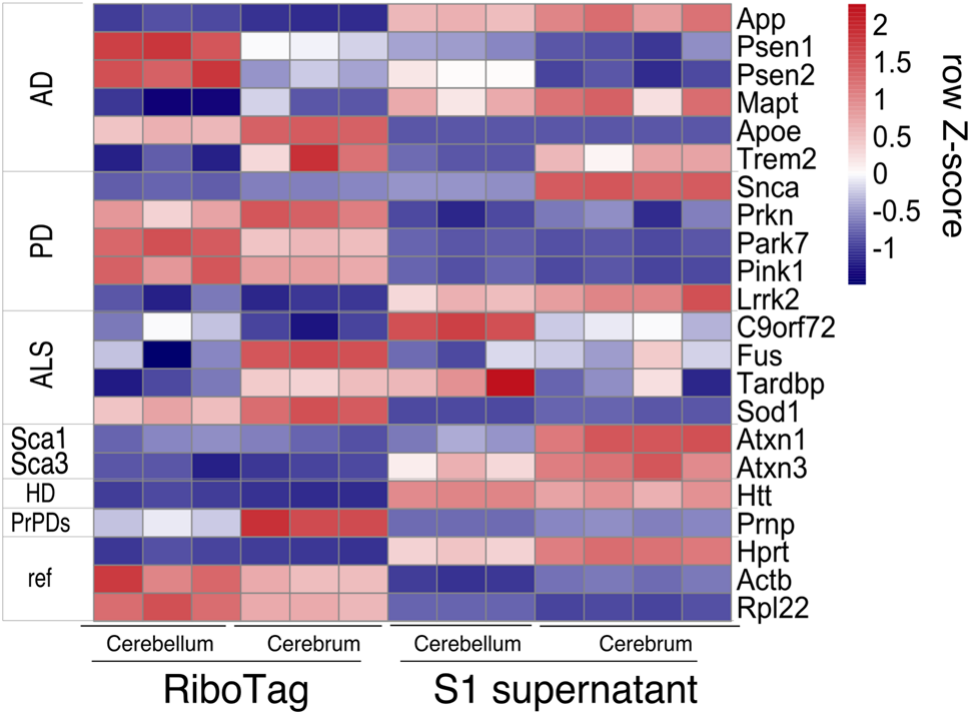
Expression of ND-related genes across the cerebellum and the rest of the brain in astroglia (RiboTag) and total input samples (S1 supernatant).

We then explored these data for new biological insights by focusing on genes known to be involved in neurodegenerative diseases (NDs). NDs result in the slow degeneration of the central nervous system and their late onset nature is especially remarkable for cases of inherited neurodegenerative diseases where the disease-causing protein is expressed throughout life. They are marked by death of neurons and morphological changes of astrocytes, sometimes becoming dysfunctional^59^. We therefore examined the expression pattern of a manually curated list of the most prominent genes linked to neurodegenerative diseases, and observed many to be unequally expressed across brain regions and cell types (Fig. 6). The mRNA from the causative gene in prion diseases, Prnp, displayed a relative enrichment in astrocytes in the cerebrum (Fig. 6) consistent with data from a Prnp reporter mouse line^60^. Interestingly, three mRNAs linked to polyglutamine diseases, Atxn1, Atxn3 and Htt, appeared to be depleted from astrocytes and therefore primarily neuronally expressed, consistent with neurons preferentially producing the disease related protein aggregates. Also, HPRT, a housekeeping enzyme, was previously used as an ectopic carrier of polyglutamine and similarly demonstrated a preferential expression in non-astroglial cells, potentially explaining the aggregation of polyglutamine-HPRT specifically in neurons and the severe phenotype observed in that model^61,62^. In contrast to these examples of monogenic diseases, amyotrophic lateral sclerosis (ALS), Parkinson’s (PD) and Alzheimer’s (AD) diseases are linked to multiple genes. Interestingly, for a given disease, some genes appear to be preferentially expressed by CA and/or BG whereas other genes appeared to be preferentially expressed by other cells. One striking example includes amyloid precursor protein (App) and presenilin 1 (Psen1), both linked to AD. Psen1 is the key component of gamma secretase, a membrane localized protein cleaving complex involved in notch pathway signaling and the generation of toxic fragments of App^63,64^. Surprisingly, App was depleted in IPs and presumably preferentially expressed by neurons, whereas Psen1 was highly enriched specifically in BG, indicating that the expression pattern of App is determined by factors besides Psen1. PD related genes also displayed contradictory expression patterns where alpha synuclein (Snca, known to be neuron specific) was depleted in CA, while Prkn, Park7 and Pink1 were enriched in astrocytes of both regions. Likewise, of genes associated with ALS, Fus was enriched in CA whereas C9orf72 was depleted in BG. Thus, multiple genes causative of the same ND often have remarkably different expression patterns. Finally, we noted that the mRNA encoding RiboTag, Rpl22, appeared to be relatively enriched in IP fractions. This prompted us to examine the expression pattern of other ribosomal proteins.

**Figure 7:**
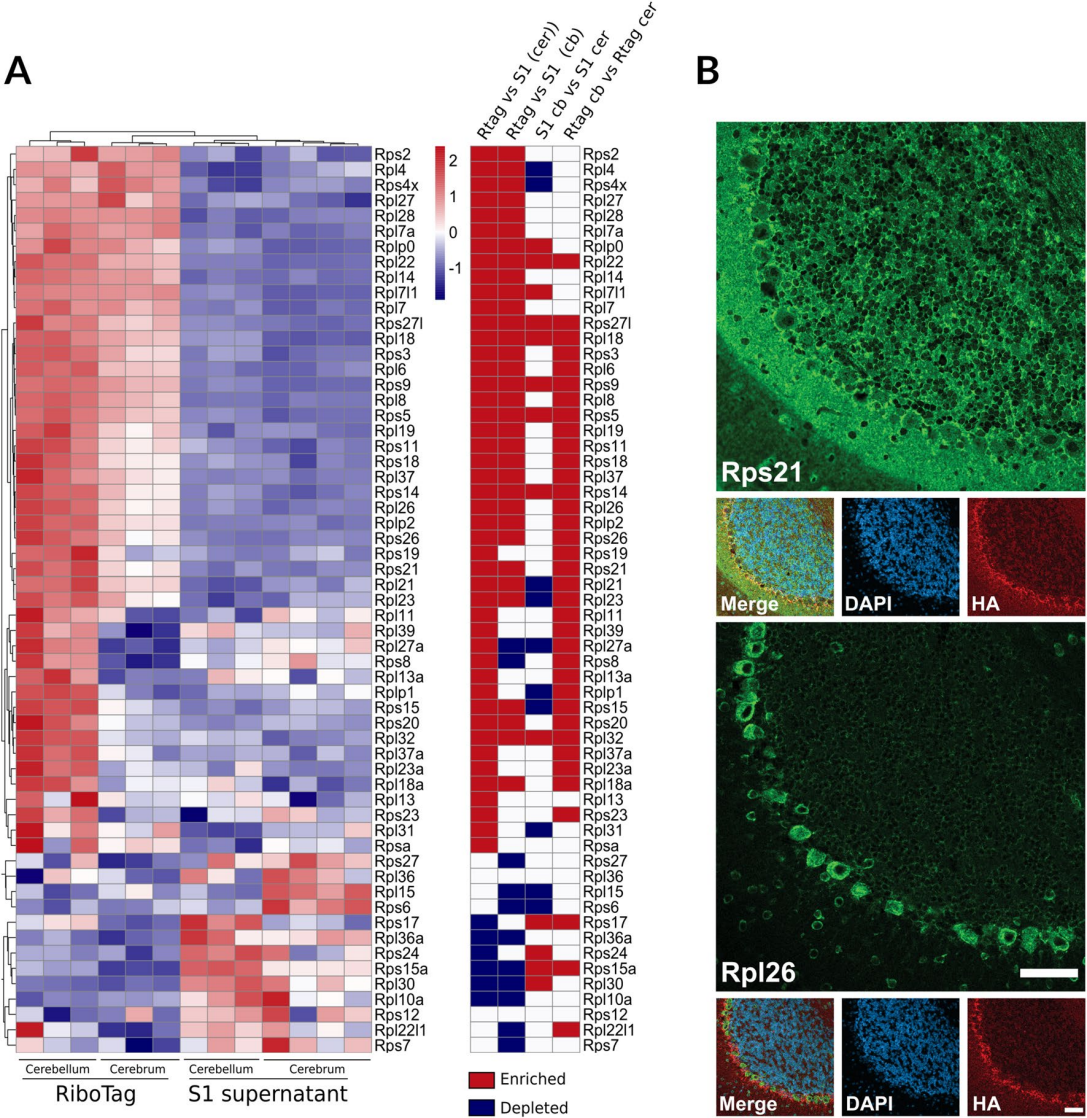
(**A**) Heatmaps of FPKM values for significantly (FDR < 0.05) enriched and depleted (RiboTag vs input) ribosomal proteins in cerebellum and cerebrum. (**B**) Immunofluorescence shows disparity between Rps21 and Rpl26 in the cerebellum. Scalebar = 50 μm.

Ribosomes are large protein synthesizing machines highly conserved across all kingdoms of life, consisting of four ribosomal RNAs and approximately 80 proteins in eukaryotes. In textbooks they are portrayed as being uniform and primarily differing only in respect to regulatory phosphorylation. However, the hypothesis that ribosome heterogeneity may function as a new layer of gene expression control is steadily gaining attention^65,66^. We therefore examined the expression profile of all the core ribosome proteins. Surprisingly, we observed a non-uniform distribution of mRNAs encoding ribosomal proteins (Fig. 7A). The vast majority of ribosome protein encoding mRNAs were more highly represented in IP fractions, suggesting that the synthesis of ribosomes commands a higher proportion of translational output for astrocytes compared to other brain cells. Interestingly, some appeared to be enriched specifically in BG (e.g., Rps8 and Rpl39), whereas others (e.g., Rps6, Rpl30 and Rpl10a) appeared to be depleted in BG (Fig. 7A). Since ribosome protein levels are not always correlated with their mRNA levels^67^, we stained brain sections with antibodies against various ribosome proteins. We used Slc1a3-RiboTag brain sections so the RiboTag protein could function as a convenient histological marker for BG, the cells our RiboTag samples were specifically captured from, and the rat derived HA antibody we commonly use could complement the available ribosomal protein-specific antibodies that are typically from rabbit. These experiments revealed that Rps21 was prominently expressed throughout the cerebellum, especially in BG but less prominent in Purkinje neurons (Fig. 7B). In contrast, Rpl26 was prominently expressed by Purkinje neurons but less so by other cells including BG (Fig. 7B). This striking difference in distribution of the proteins was unexpected as the mRNAs suggested they share a similar expression pattern (Fig. 7A) and indicates that factors beyond mRNA levels control the distribution of ribosome proteins. In summary, these translatome data provide a resource to study the characteristics of BG that make them unique.

## Discussion

Minimization of genetic variability by inbreeding has become a gold standard in research using mouse models. Selection of mice from highly uniform populations increases the statistical power, thereby making experiments more sensitive and efficient, enabling a reduction in the number of replicates and mouse lives needed. The desire for using inbred mouse lines has led to many being available exclusively on a B6 background. However, the high inter-individual variability we observed in mice backcrossed to B6^14,15^ and here in inbred B6 could confound experimental results, especially those involving high content gene expression analyses. Although our behavioral study excluded males, it is not unusual to observe B6 males jumping or running excessively, though we have not witnessed this in S4 males. For this reason, it is worthwhile considering alternative genetic backgrounds. Our results do not indicate that B6 are abnormal. In fact, high activity and tendency to explore, if interpreted as eagerness to escape captivity, is how wild mice ought to behave. In this sense, S4 mice might be considered abnormal due to their docile character and low activity levels. Although they were historically bred to study testicular cancer^68^, we are aware of only a single case of spontaneous testicular tumor formation during several years of work with multiple lines in this genetic background, often aged up to 2 years. Importantly, most embryonic stem cell lines used for the generation of gene-targeted mice are derived from 129 strains, especially S4, and the S4 strain is a robust breeder, so it is a logical complement to B6 strains. Therefore, the highly homogeneous and calm nature of S4 is desirable for studies of gene expression which prompted us to establish several mouse lines in this alternative background (Table 1). A mouse model of AD is already available in the S4 background (https://www.jax.org/strain/031988) and we have backcrossed to S4 a knock-in mouse model of HD^50^.

Astrocytes are essential for normal brain function and play crucial roles in synaptic plasticity^69^, removing extracellular waste^70^, and actively participate in neural processes controlling behavior^71^. Disruption of astrocyte function contributes to a variety of diseases^72–75^. Therefore, a steady need for tools to access astrocytes in vivo exists. However, several features of astrocytes make them notoriously difficult to genetically target^76–78^. First, they make less total protein, and the half-lives of their proteins are typically shorter than in neurons^79^. As a result, the levels of effector proteins expressed in astrocytes are often insufficient. Second, several marker genes specific for adult astrocytes are also expressed in NPCs during development^37,38^, many of which become neurons. This likely explains why many of the earliest lines developed based on constitutive activity of Cre from mouse or human GFAP promoter elements targeted neurons as well^80–82^ . Fortunately, this can often be circumvented with CreERT2. The Slc1a3 gene was previously employed to drive CreERT2 using a BAC method. BAC transgenic mice have important strengths, such as being easier to develop and can provide much higher expression levels than the native gene. However, since each integration occurs in a different genomic location and with various copy numbers, variable activity may occur if native elements of the genomic integration site compete with the regulatory elements carried by the transgene^42^. Indeed, the recombination efficiency and specificity of a BAC-GLAST-Cre line (using Slc1a3’s alternative name) was variable depending on the founder line employed^32^. Moreover, some BAC-GLAST-CreERT2 mice showed significant recombination in neurons, in spite of utilizing the same promoter elements, possibly due to multiple integrations, or integrations in the vicinity of neuronal enhancers and, consequently ectopic expression of Cre^83^. Notably, that line also targeted NPCs. Since NPCs express Slc1a3 it is likely that our Slc1a3-2A-CreERT2 knock-in line will also be active there but consequences of this might be avoided by limiting the time between activation and sacrifice. We did not test the specificity of our line in the peripheral nervous system or other organs but efforts to do so will be well placed.

Another potential problem with BAC transgenics is that the invading transgene greatly alters the integration site. For example, besides inserting large (often hundreds of kilobases) fragments of ectopic DNA that can interrupt native genomic neighborhoods^42^, it is not uncommon for large deletions to also occur at the site of integration^41^. Moreover, BACs typically carry additional genetic elements. For example, in BAC Aldh1l1-CreERT2 mice the transgene appears to also carry genes encoding a protein (Slc41a3), a miRNA, and a long non-coding RNA^84^. Slc41a3 is a magnesium transporter highly expressed in cerebellar Purkinje cells. Since magnesium is a modulator of neurotransmitter receptors^85,86^ and cofactors and/or allosteric modulators of enzymes^87,88^, overexpression of Slc41a3 might alter brain homeostasis. The effects of overexpressing the miRNA and long non-coding RNA are more difficult to predict but should not be ignored.

Fortunately, such problems can be avoided with gene targeting. A previous gene-targeted mouse line using Slc1a3 resulted in the complete inactivation of the host gene^31^. Although Slc1a3 knock-out mice are viable, they display some differences from wild types^89^. We attempted to avoid this through use of the 2A system but the residual 2A peptide on Slc1a3 appeared to reduce the steady state level. Such an effect has been demonstrated in cultured cells^90^. The relative counts of mRNA sequencing reads mapping to Slc1a3 and CreERT2 coding sequences in heterozygous mice indicated that mRNA levels are only mildly affected and the reduced expression is caused by a post-transcriptional effect. Furthermore, we have not determined if Slc1a3 protein carrying the 2A peptide is functional. This unexpected observation may call for caution when applying the 2A peptide method to express two proteins from a single mRNA. Nonetheless, we maintain the line as homozygotes, which appear, behave and reproduce like wild-type S4, and any phenotypic effects for Slc1a3-2A-CreERT2 mice will likely be negligible since heterozygotes are used for experiments.

A recently reported Aldh1l1-T2A-CreERT2 knock-in line was shown to have perfect specificity and very high efficiency, 50 to 80%, depending on the region and induction protocol^91^. The gene-targeted Slc1a3 Cre-ERT2 line mentioned above^31^ was tested in parallel and both lines had very favorable and similar performance^91^, consistent with the data we present here. Since a given Cre line can yield very different recombination efficiencies of floxed alleles at different loci, likely due to differences in expression intensity and therefore chromatin states^92^, we employed reporter lines at two different genomic loci. PC::G5-tdT was inserted 1 kb downstream of the Polr2a gene whereas RiboTag was built into the Rpl22 gene. Both are highly and ubiquitously expressed genes which may have benefited our study with good induction for both. Indeed, the evaluation of the Aldh1l1-T2A-CreERT2 knock-in line employed a 7-day induction protocol followed by 2 weeks of expression whereas we employed shorter induction strategies for RiboTag that yielded similar induction rates. Thus, Aldh1l1-T2A-CreERT2 knock-in mice may yield an even higher efficiency if paired with an alternative reporter line. Unfortunately, the genetic background of the Aldh1l1-T2A-CreERT2 knock-in line was not clearly described.

We validated the S4 Slc1a3-2A-CreERT2 line with RiboTag translatome data from two brain regions. Besides establishing that RiboTag was activated specifically in astroglia, these data revealed new information. First, the comparison of translatomes of BG to CA revealed that some genes thought to be markers for astrocytes or other cells are selective for one brain region but not the other, expanding the concept that astrocytes are highly diverse. Second, our data may be interpreted as supporting the hypothesis of ribosomal specialization^93^. Although it is controversial^94^, the concept of specialized ribosomes posits that the stoichiometry of ribosome proteins on ribosomes results in preferential translation of a subset of mRNAs in the translatome^95,96^. While our observations do not provide conclusive proof for ribosomal specialization, they indicate that conditions needed for specialized ribosomes exist in the brain. Third, we found that genes causative of a single ND had disparate expression patterns. For example, Psen1 is important for processing App into disease forms that cause AD, but their expression patterns are different (Fig. 6). Likewise, genes involved in PD and ALS also demonstrated contradictory expression patterns (Fig. 6). Therefore, selective vulnerability to neurodegenerative disease, the concept where specific brain regions are most severely targeted in a disease-specific pattern is not simply determined by expression patterns of the disease-causing proteins^97^. Beyond neurodegenerative diseases, this mouse line may prove useful in other fields of biomedical research, for example in vivo screens of glioma^98,99^.

Finally, we thought it would be interesting to compare the translatomes of B6 and S4 mice using the same Slc1a3-2A-CreERT2 construct. However, this would require either extensive backcrossing or recreating the strain directly in B6 ESCs and also acquiring RiboTag in B6. Considering our commitment to using a genetic background other than B6, the line would subsequently be of little use for our research program, and this interesting comparison remains unattempted.

S4 Slc1a3-2A-CreERT2 mice are available at the European Mouse Mutant Archive EM:11,807 and the RiboTag gene expression data can be accessed at https://jaws.research.liu.se/resources.html.

## Methods

### Ethical statement

Ethical permissions for this work were granted by the Landesamt für Natur, Umwelt und Verbraucherschutz Nordrhein-Westfalen, permission #84-02.04.2012.A192, #84-02.04.2017.A016 and #84-02.04.2016.A442, or the Massachusetts Institute of Technology Committee on Animal Care #0705-044-08. All experimental procedures were performed in accordance with the internal regulations of the DZNE or MIT. This study was performed in compliance with the ARRIVE guidelines.

### Mouse line generation

Targeting vector was constructed in the pBlueScript backbone using combination molecular biology methods and gene synthesis. Homology regions were PCR-amplified from 129S4 genomic DNA. Cre-ERT2 sequence was pCAG-CreERT2 was a gift from Connie Cepko (RRID: Addgene_14797). The targeting vector is available at Addgene (RRID: Addgene_129409). Gene targeting was done in J1 ES cells (ATCC) following methods described previously^100^. The stop codon of Slc1a3 was targeted with Cas9 by the double nickase strategy^101^, using the pX335 construct (a gift from Feng Zhang, RRID: Addgene_42335), bearing GTT CTC TGT CCG TCT ACA TCT and GCT TTC TTA AGC ACC AA GTG T sgRNA protospacers. Targeting was verified by PCR using LongAmp HotStart polymerase (NEB) using primers listed in Table S1.

Gene targeted cells were injected into C57Bl/6N blastocysts using established methods. A male chimera transmitting the ES cell genome to progeny was identified by first breeding to C57Bl/6N females, and subsequently bred to S4 females expressing Flpo recombinase (Jax line #007844)^43^ to simultaneously remove the selection cassette and establish the line in S4 background. This F1 generation was bred two more times to S4 mice to remove the Flpo transgene and any spontaneous mutations arising from culturing or storage of the ES cells. In order to dampen effects of genetic drift, every 5th or 6th generation we cross in fresh S4 for two generations before intercrossing and maintaining as homozygotes. RiboTag mice were acquired from Jackson labs (Jax line #011029), with a predominately B6 background. They were backcrossed to S4 for 10 generations, periodically guided by SNP analysis for choosing breeders. A final SNP genotyping analysis (Envigo RMS, Inc, Indianapolis, USA) measuring 347 SNPs that discriminate S4 from B6 revealed only 1 residual B6 SNP, indicating the genetic background of the line is approximately 99.7% S4. For backcrossing work, S4 mice were obtained from Jackson labs (Jax line# 009104). For behavioral experiments, wild type B6 (C57Bl/6NTac) mice were acquired from Taconic and wild type S4 mice were acquired from the Whitehead Institute/Massachusetts Institute of Technology, where this strain originated, and both strains were bred in house less than 5 generations prior to inclusion in experiments.

### Automated mouse behavioral analysis

Video recordings were performed as described previously^14,15^ at 6, 8, 10, 12, 14, 16, 18, and 20 months of age on female mice at the Whitehead Institute for Biomedical Research, Cambridge, Massachusetts. Since video recordings must be of mice in individual cages and returning males into group housing may induce fighting, males were excluded from the study. Mice were kept in a specific pathogen free facility in standard individually ventilated cages with shredded wood bedding and most cages holding 4 mice but some holding 3 or 5, always littermates. They had free access to standard mouse chow and autoclaved water and lived in a 12 h light:dark cycle. Mice were separated into individual cages 1 h prior to the transition from light to dark and video recordings were started immediately. Following the 24-h recordings mice were recombined with their original cage mates. Videos were analyzed with Home Cage Scan software (CleverSys Inc.) followed by a customized downstream pipeline^14,35^. The behavior “urinate” is not reliably scored so was disabled in this analysis. Otherwise, the heatmap displays all metrics studied. Two-tailed Wilcoxon rank sum (non-parametric) test was applied and a P-value < 0.05 was considered significant. Multiple tests corrections were not deemed necessary since nearly all metrics showed large differences. For most mice weight was measured monthly. In a first cohort of mice weight measurements were initially stopped when it was noticed that B6 mice were highly variable. In the second cohort mice were weighed at 18 months. Violin plots were generated using ggplot2 (https://ggplot2.tidyverse.org). Horizontal lines represent medians.

### Tamoxifen injections

To induce expression of the GCaMP5g-tdTomato reporter, mice were injected i.p. with 5 μl/g body mass with an emulsion of 20 mg/ml tamoxifen (Sigma, #T5648) in sunflower oil:ethanol mix (10% ethanol). Sunflower oil was from Sigma (#47123). Three months old mice were injected for five consecutive days, followed by imaging 3 weeks later. To induce expression of the RiboTag reporter, mice were injected with 10 μl/g body mass with an emulsion of 10 mg/ml tamoxifen (Sigma, #T5648) in sunflower oil:ethanol mix (10% ethanol). Injections were done daily for three total injections, and mice were sacrificed 4 days after the final injection at approximately 2 months of age for RiboTag studies. The use of different induction procedures for each reporter was chosen based on successful experiments with these reporters using other Cre lines.

### Cranial window preparation

Hippocampal window surgery was performed 4 weeks before imaging as described previously^55^. Mice were anesthetized with isoflurane (induction, 3%; maintenance, 1–1.5% vol/vol; Virbac), and body temperature was maintained with a heating pad (37 °C). Mice received buprenorphine (0.1 mg/kg; Reckitt Benckiser), dexamethasone (0.2 mg/kg; Sigma Aldrich #D1159) and cefotaxime (2 g/kg; Fisher Scientific #15219946). After fixation in a stereotaxic frame, the skin was removed under sterile conditions and a craniotomy (diameter, 3 mm) above the right somatosensory cortex (coordinates: AP – 1.9 and ML + 1.25 relative to bregma) was created with a dental drill. The dura was removed, and the somatosensory cortex was removed with a 21G needle attached to a 20-ml syringe with a flexible tube. When the external capsule of the hippocampus was reached, the alveus was carefully exposed using a 27G needle. Subsequently, a metal tube (diameter, 3 mm; height, 1.5 mm) sealed with a glass coverslip (diameter, 3 mm) was inserted, and the upper tube edge was glued to the skull bone using dental cement. The remaining exposed surface of the skull bone was sealed with dental cement, and a custom-made metal bar was glued next to the metal tube. Mice received buprenorphine (0.1 mg/kg) for 3 days after surgery.

For cortical window preparation, mice were anesthetized with isofluran (induction, 3%; maintenance, 1–1.5% vol/vol), and body temperature was maintained with a heating pad (37 °C). Mice received buprenorphine (0.1 mg/kg), dexamethasone (0.2 mg/kg) and cefotaxime (2 g/kg). After the mice were fixed in a stereotactic frame, the scalp was removed and a craniotomy (diameter, 3 mm) was created above the right somatosensory cortex using a dental drill. The surface was rinsed with sterile saline, a coverslip (diameter, 3 mm) was inserted and glued with dental cement, and a headpost (Luigs & Neumann) was glued adjacent to the cortical window. Mice received buprenorphine (0.1 mg/kg) for 3 days after surgery. Schematic images of cranial windows were prepared using BioRender (https://biorender.com).

### In vivo two-photon microscopy

Mice implanted with hippocampal windows were imaged using an upright two-photon microscope (Trim ScopeII; La Vision) with a 16 × objective (NA 0.8, Nikon LWD16x) and three non-descanned detectors with two bandpass filters (617/73, 525/50 nm) and one long-pass filter (550 nm). Mice were imaged anesthetized (with isoflurane; 1–1.5% vol/vol) and awake on a treadmill (Luigs & Neumann). For hippocampal imaging, the same group of mice was imaged under awake and anesthetized conditions. Fluorophores were excited at 920 nm using a Titan Sapphire (Ti:Sa) laser (Chameleon Ultra II; Coherent; 140-fs pulse width, 80-MHz repetition). XY time-lapse series of astroglial calcium activity (256 × 256 μm; 256 px; pixel dwell time, 1.62 μs) were subsequently recorded for 10 min at 3.61 Hz at a depth of 100– 200 μm beneath the hippocampal surface. Mice implanted with cortical windows were imaged awake on a treadmill using an upright two-photon microscope (SP8 DIVE (Deep In Vivo Imager); Leica) with a 16 × objective (NA 0.8, Nikon LWD 16x) and hybrid detectors (HyD) with two filters (500/550, 560/620 nm). Fluorophores were excited at 920 nm using a Spectra Physics InSight DS + laser. XY time-lapse series of the astroglial calcium activity (256.28 × 256.28 μm; 256 px; pixel dwell time, 975 ns) were recorded for 10 min at 3.82 Hz at a depth of 100–200 μm beneath the cortex surface. Laser power below the objective was kept between 20 and 40 mW to minimize laser-induced artifacts and phototoxicity.

### Calcium imaging data analysis

Calcium imaging data were stabilized using a custom-written Lucas-Kanade algorithm^102^ in Matlab R2018a (MathWorks). Regions of interests (ROIs) representing astroglial microdomains were defined by fluorescence changes over time in GCaMP5g-expressing astrocytes using a custom-written macro (National Institutes of Health) in ImageJ 1.50i^103^. Time-lapse data for each ROI were normalized, smoothed, and peak candidates were detected with a hard threshold. Detection and classification of fluorescence peaks over time was performed with a custom-written algorithm in Python. Mean fluorescence data were first normalized by a robust z-score calculated per ROI over the whole time-lapse series. Normalized data were then smoothed with a Gaussian filter, and all maxima above the threshold were selected as peak candidates. Peak candidates were defined by its ROI and the timepoint of peak maximum. Peak amplitude and full duration at half maximum (FDHM) were determined for each peak candidate. Each time-lapse series was plotted together with the respective video file for visual inspection and verification.

### RNA-seq and data analysis

RiboTag-purified and total RNA samples were sequences at Atlas Biolabs. Library was prepared with TruSeq Stranded mRNA protocol and samples were indexed using TruSeq RNA CD Index Plate. Libraries were QC-ed and quantified on TapeStation system (Agilent) and 50 bp single reads were obtained from HiSeq2500 sequencer (Illumina). Demultiplexing was done using standard Illumina pipeline and reads were QC-ed using FastQC. Reference (GENCODE GRCm38) was indexed, and the reads were aligned with BBmap using the following parameters: in = in.fq out = out.bam qtrim = t usequality = t minaveragequality = 0 local = f strictmaxindel = f xstag = us maxindel = 100,000 intronlen = 10 ambig = toss threads = 8. BAM files were indexed and sorted with Samtools v1.9 and exon counts were obtained with summarizeOverlaps() R function. RPKM values and gene enrichments/depletions were computed using DEseq2 R package^104^. Heatmaps were generated using pheatmap() R package. Statistical significance of gene list overlaps (Venn diagrams) was computed with hypergeometric test using phyper() R function. GO analysis was done with WebGestalt^105^. Violin plots were generated using ggplot (https://ggplot2.tidyverse.org/). Gene expression data can be browsed via a Shiny app at https://jaws.research.liu.se/resources.html and raw data is available at GEO database (GEO GSE145484).

### Immunohistochemistry

#### GCaMP5g, tdTomato and Sox9 staining (Fig. 3)

Cre recombination was induced by tamoxifen injection in 3-month-old mice. Mice were sacrificed by carbon dioxide asphyxiation, and one hemisphere was fixed in 4% paraformaldehyde for 1 day, stored in sucrose (15% and 25%), and embedded in Tissue-Tek (Fisher Scientific #12678646). Sagittal sections (30 μm) were obtained using a cryostat (Thermo Fisher) and mounted onto slides. Brain sections were blocked with 10% normal goat serum (Vector Labs) and 0.3% Triton X-100 (Sigma) in PBS for 1 h. Subsequently, brain sections were incubated with chicken anti-GFP (1:500; Abcam, RRID: AB_300798) and rabbit anti-SOX9, 1:2000 (Millipore, RRID: AB_2239761) in 5% normal goat serum and 0.05% Triton X-100 (Roth #3051.3) in PBS overnight at 4 °C followed by 3 × rinsing with 5% normal goat serum (Biozol #VEC-S-1000) in PBS for 5 min. Sections were incubated with secondary antibodies from goat (anti-chicken Alexa Fluor 488 and anti-rabbit Alexa Fluor 647, 1:1000 (Thermo Fisher) in PBS and 0.05% Triton X-100 for 3 h at room temperature, rinsed, and mounted in Fluoromount-G (Southern Biotech). Images were acquired using either a confocal laser-scanning microscope (LSM 700; Zeiss) with a 20x (NA 0.8) objective and a slide scanner (Axio Scan.Z1; Zeiss) with a 10 × objective (NA 0.45), with the following filter settings: LSM700, 490–555 BP, 640 LP, 560 LP; AxioScan.Z1, 470/40 BP, 525–50 BP, 587/25 BP, 647/70 BP, 640/30 BP, 690/50 BP. The same image acquisition settings were used for each staining. Immunohistochemical data were quantified using a customized pipeline that includes the automated identification of the Sox9 positive cell nuclei and GCaMP positive cell cytoplasm using CellProfiler.

#### Cell type marker stainings (Fig. 4)

Mice were sacrificed by carbon dioxide asphyxiation, then brain hemispheres were emersion fixed in 10% buffered formalin for two days. After cryoprotection in 30% sucrose in PBS, 40 µm coronal cryosections were taken and sections were stored until use in 50% 0.1 M PBS, 30% ethylene glycol, and 20% glycerol at – 20 °C. Sections at the level of AP-1.46^106^ were selected, and double incubated overnight at 4 °C in primary antibodies against HA, 1:200 (clone: 3F10, Roche, AB_2314622) and the respective marker antibody. Primary antibodies (with dilutions): OSP, 1:200 (Abcam, RRID: AB_2276205), NeuN, 1:500 (Millipore, RRID: AB_2298772), Sox9, 1:2000 (Chemicon, RRID: AB_2239761), GFAP, 1:500 (Dako, RRID: AB_10013382), Iba1, 1:100 (Wako, RRID:AB_839504). Secondary antibodies (dilutions 1:1000): anti-rat IgG to detect 3F10 (Jackson ImmunoResearch 712–165-153 or 712–545-153) and appropriate second antibody (mouse: 715–545-151; rabbit: 711–225-152 or 711–585-152). Micrographs of the CA1-region of the hippocampus and deep layers of cortex immediately above CA1 were taken with a confocal microscope (LSM 700, Zeiss).

#### Ribosomal proteins (Fig. 7)

At 6 weeks of age mice were sacrificed by carbon dioxide asphyxiation and brains removed and emersion fixed in 10% formalin for 2 days. Brains were then processed through zylenes and paraffin and placed in cassettes. Cassettes were cut into 4 µm thick sections. Sections were dewaxed in histolab clear (Histolab #14250) and rehydrated in graded dilutions of ethanol, 100–0% (each 5 min). Epitope retrieval was performed with a steamer in 0.01 M citrate buffer, pH 8, for 20 min and cooled down for 15 min at RT before incubated in dH_2_O for 5 min. Auto fluorescence was quenched using the TrueBlack Lipofuscin Autofluorescence Quencher (Biotium #23007) according to the manufacturer’s protocol. Sections were incubated in blocking buffer (PBS with 2.5% NHS) for 30 min prior to addition of primary antibody solution for 2 h at RT. Double stains were performed using a mix of the two primary antibodies in blocking buffer simultaneously. Then, sections were washed in PBS for 3 × 5 min. After the last wash step secondary antibodies diluted in blocking buffer were added for additional 30 min incubation, RT. A second wash with PBS for 3 × 5 min, RT, was conducted prior to the use of a second auto fluorescence quenching kit (Vector TrueVIEW autofluorescence quenching kit, Vector Laboratories, # SP-8400). Finally, sections were washed in PBS for 5 min, incubated with DAPI (conc. 0.1 µg/ml) for 5 min and washed in PBS for 5 min. Mounting was performed using Vectashield vibrance antifade mounting media (Vector Laboratories, #H-1700). Sections were imaged on the confocal microscope LSM 800 (Zeiss) using a 20 × objective. Primary antibodies: HA-tag, 1:100 (Roche, RRID: AB_390919), RPS21 1:100 (Bethyl, RRID: AB_2631466), RPL26 1:100 (Cell Signaling, RRID: AB_10698750). Secondary antibodies (each used at 1:500): Alexa Fluor 647-goat-anti-rat (Thermo Fisher), Alexa Fluor 488-donkey-anti-rabbit (Jackson ImmunoResearch, RRID: AB_2313584).

### RNA in situ hybridization

FFPE sections were prepared as for ribosomal staining sections. Prior to dewaxing sections were incubated in an oven at 50 ∘C for 1 h. Afterwards we followed the ACDbio protocol using RNAscope 2.5 HD Assay Red and the following probes: Aldh1l1: 405891, Gja1: 486191, Slc1a3: 430781.

### Immunoblotting

RiboTag supernatants were mixed with 4 × LDS (lithium dodecyl sulfate) sample buffer containing 40 mM DTT and denatured at 70 °C for 10 min prior to loading on 10% NuPAGE Novex midi gels (Thermo Fisher). Gels were run using MES [2-(*N*-morpholino)ethane sulfonic acid] running buffer at 150–160 V (110 V for the first 15 min) and then were electro-transferred to nitrocellulose membrane (Bio-Rad), submerged in transfer buffer (20% methanol, 25 mM Tris–Cl, 0.19 M glycine), using a Criterion transfer tank (BioRad), at 0.7 A, for 70 min. Membranes were blocked for 20–30 min at room temperature (RT) in 5% powdered milk in PBS-T (PBS with 0.05% Tween-20) and then incubated with primary antibody diluted in blocking buffer overnight at 4 °C. Next, blots were washed 4 × with PBS-T and incubated with secondary antibody for 30–60 min at RT, followed by 5 × PBS-T washes and imaging with the Li-Cor Odyssey imaging system (Li-Cor). For re-probing, membranes were stripped for 10 min with Re-Blot strong solution (EMD-Millipore), followed by extensive washing and re-blocking. Primary antibody: anti-Slc1a3 (dil. 1:2000, CST, clone D20D5, RRID: AB_10694915), rabbit-anti-β-actin (dil. 1:10.000, Sigma, RRID: AB_476697). Secondary antibodies: donkey-anti-rabbit IRDye 680RW (dil. 1:10.000, Li-Cor, RRID: AB_10706167), donkey-anti-mouse IRDye 800CW (dil. 1:20.000, Li-Cor, RRID: AB_1850023). Dimers are paired proteins detected in western blot experiments and are noted for multiple commercial antibodies against Slc1a3 protein.

### Translatome purification with RiboTag

We previously optimized our RiboTag protocol to increase its specificity^25^. For RiboTag experiments mice were sacrificed at 50 days of age. Mice were sacrificed by carbon dioxide asphyxiation, brain was removed, and olfactory bulb was cut out, and then cerebellum separated from cerebrum. Deep frozen brain tissue was used to prepare homogenate in Polysome Buffer (PSB) containing 50 mM Tris (pH 7.5), 100 mM KCl, 12 mM MgCl2 and 1% Nonidet P-40, 1 mM DTT, 100 U/ml RiboLock RNAse inhibitor, 100 µg/ml cyclohexamide and 1 tab/5 ml SigmaFast protease inhibitor cocktail with EDTA (Sigma). One hemisphere was used per replicate. Homogenates were prepared with 1 ml and 2 ml of PSB was used per cerebellum (both hemispheres were pooled) and cerebrum (the remaining part of the brain left after removal of cerebellum and olfactory bulb, one hemisphere) respectively, in Potter–Elvehjem homogenizers using a motorized (~ 450 rpm) pestle and then cleared by centrifugation (10.000 g / 10 min / 4 °C) to remove nuclei and cell debris to obtain the supernatant (S1). 50 μl of lysate was immediately mixed with Qiazol and purified as input. Supernatant was pre-cleared with Protein G Dynabeads (PGDB, Thermo Fisher) for 30 min at 4 °C on a rotator and S1 (1 ml for cerebellum and 1 ml for cerebrum) was incubated with 5 μg of anti-HA mAb (Roche, clone 12CA5, RRID: AB_514505) at 4 °C for 60 min on a rotator. Sample was then transferred to prepared equivalent of 37.5 μl total bead suspension PGDB (PSB-equilibrated) and incubated as above for additional 100 min. Afterwards, both Ribo-Tag and Ago-Tag beads were washed 3 × 5 min in High Salt Buffer (HSB) containing 50 mM Tris (pH 7.5), 300 mM KCl, 12 mM MgCl2 and 1% Nonidet P-40, 1 mM DTT, 50 U/ml Ribolock RNase inhibitor (Thermo Fisher), 100 µg/ml cycloheximide, 1 tab/20 ml of SigmaFast protease inhibitor cocktail with EDTA (Sigma) and additional 3 × 5 min in Extra High Salt Buffer (EHSB, identical to HSB but containing additional 300 mM NaCl). During each wash, beads were rotated gently at 4 °C. Following removal of the last wash solution, 700 μl Qiazol (Qiagen) was added and the beads were incubated for 15 min at RT with vigorous (> 1000 rpm) agitation. RNA was extracted from input and RiboTag samples using miRNeasy Micro kit (Qiagen) and eluted with 28 μl of water.

## Supplementary Information

Supplementary Video.

Supplementary Information.
