# Supplementary material for: Slc1a3-2A-CreERT2 mice reveal unique features of Bergmann glia and augment a growing collection of Cre drivers and effectors in the 129S4 genetic background
Source: Sci Rep. 2021 Mar 8;11:5412. doi: 10.1038/s41598-021-84887-2 (PMC7940647; doi:10.1038/s41598-021-84887-2)
Supplement: Supplementary file 2 — Supplementary Information. [file 41598_2021_84887_MOESM2_ESM.pdf]

TITLE

Slc1a3-2A-CreERT2 mice reveal unique features of Bergmann glia and augment a growing collection of Cre drivers and effectors in the 129S4 genetic background

AUTHORS

Lech Kaczmarczyk[1,2]
Nicole Reichenbach[2]
Nelli Blank[2]
Maria Jonson[1]
Lars Dittrich[2]
Gabor C. Petzold[2,3]
Walker Jackson[1,2*]

1. Wallenberg Center for Molecular Medicine, Department of Biomedical and Clinical Sciences, Linköping University, Linköping, Sweden
2. German Center for Neurodegenerative Diseases (DZNE), Bonn, Germany
3. Division of Vascular Neurology, Department of Neurology, University Hospital Bonn, Germany

(*) Corresponding author: walker.jackson@liu.se

Supplementary movie 1. This movie presents a snapshot of hyperactivity observed for mice in the top left (mouse 8974) and bottom right (mouse 8977) cages. Similar observations were made at many times throughout the night, and at many ages.

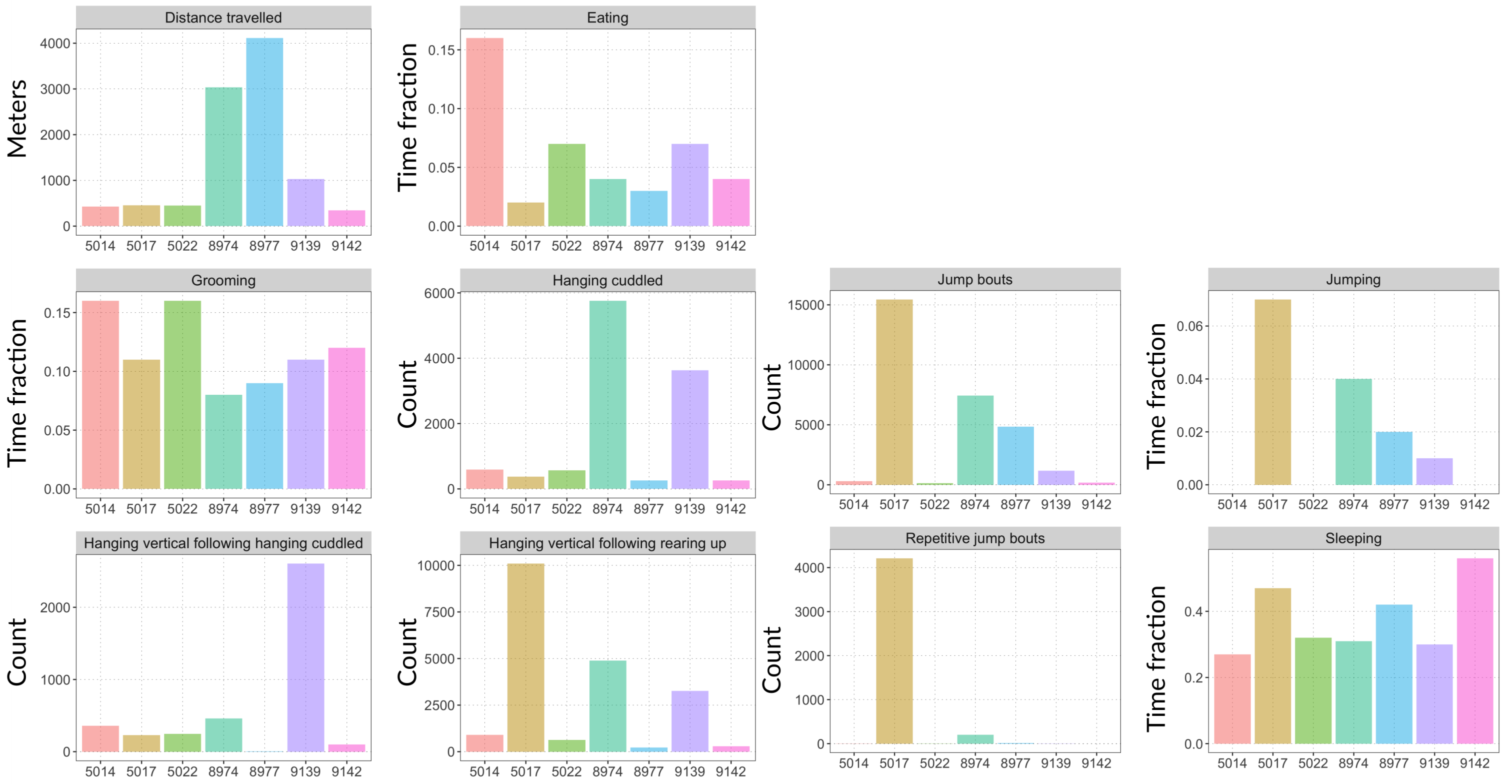

Supplementary figure 1. Automated mouse behavioral analysis data from selected individual mice depicted in the figure 1, representing most significant outliers in the B6 cohort. X-axis labels denote mouse ID numbers.

A

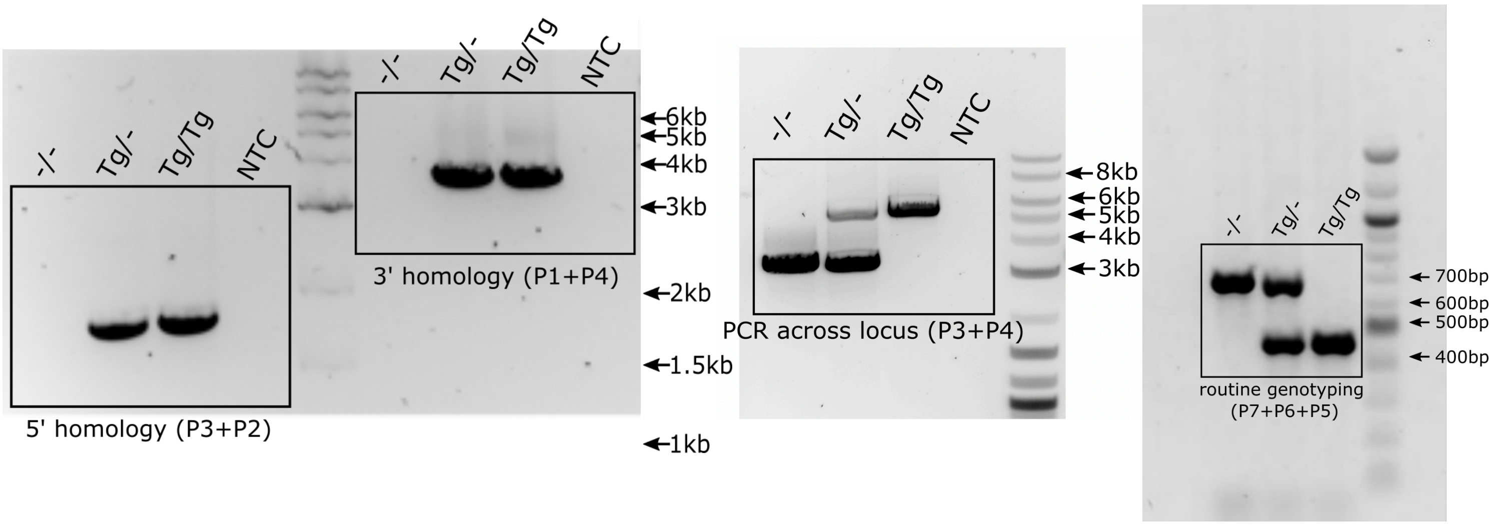

B

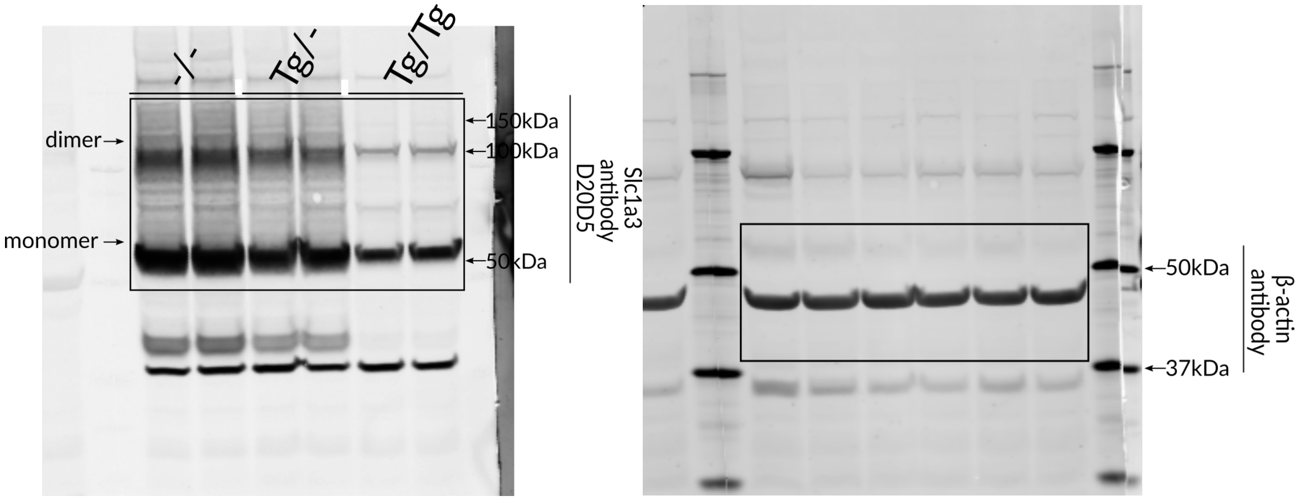

Supplementary figure 2. Uncropped images of gels and immunoblots shown in figure 2C-F (**A**) and figure 2G (**B**). Frames denote cropping boundaries. Dimers in B are commonly seen with antibodies for Slc1a3

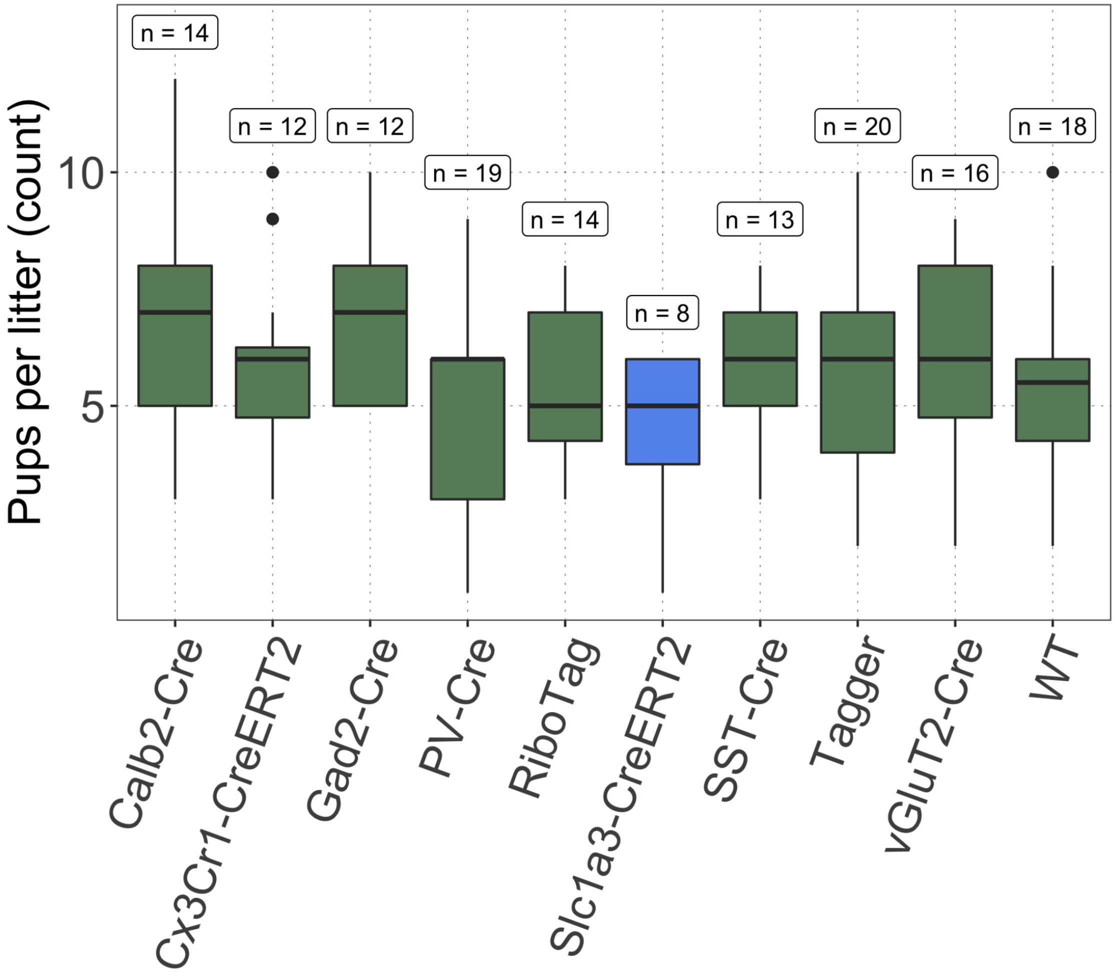

Supplementary figure 3. Box plots display the breeding outputs of different mouse lines (seven Cre driver lines and two Cre effector lines) in 129S4 background. WT = wild type. The number above each box plot refers to the number of litters analyzed.

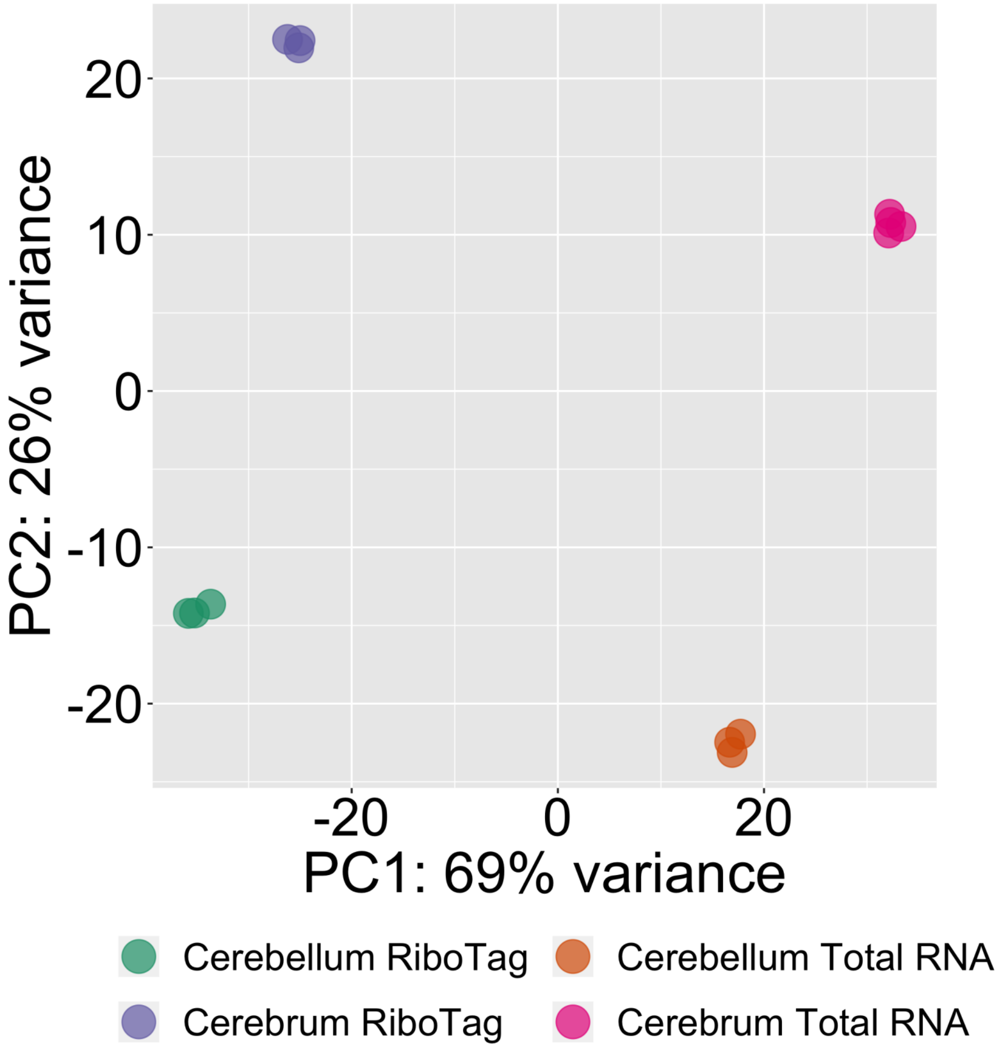

Supplementary figure 4. Principal component analysis (PCA) of RNA-seq data from RiboTag IPs and corresponding supernatants.

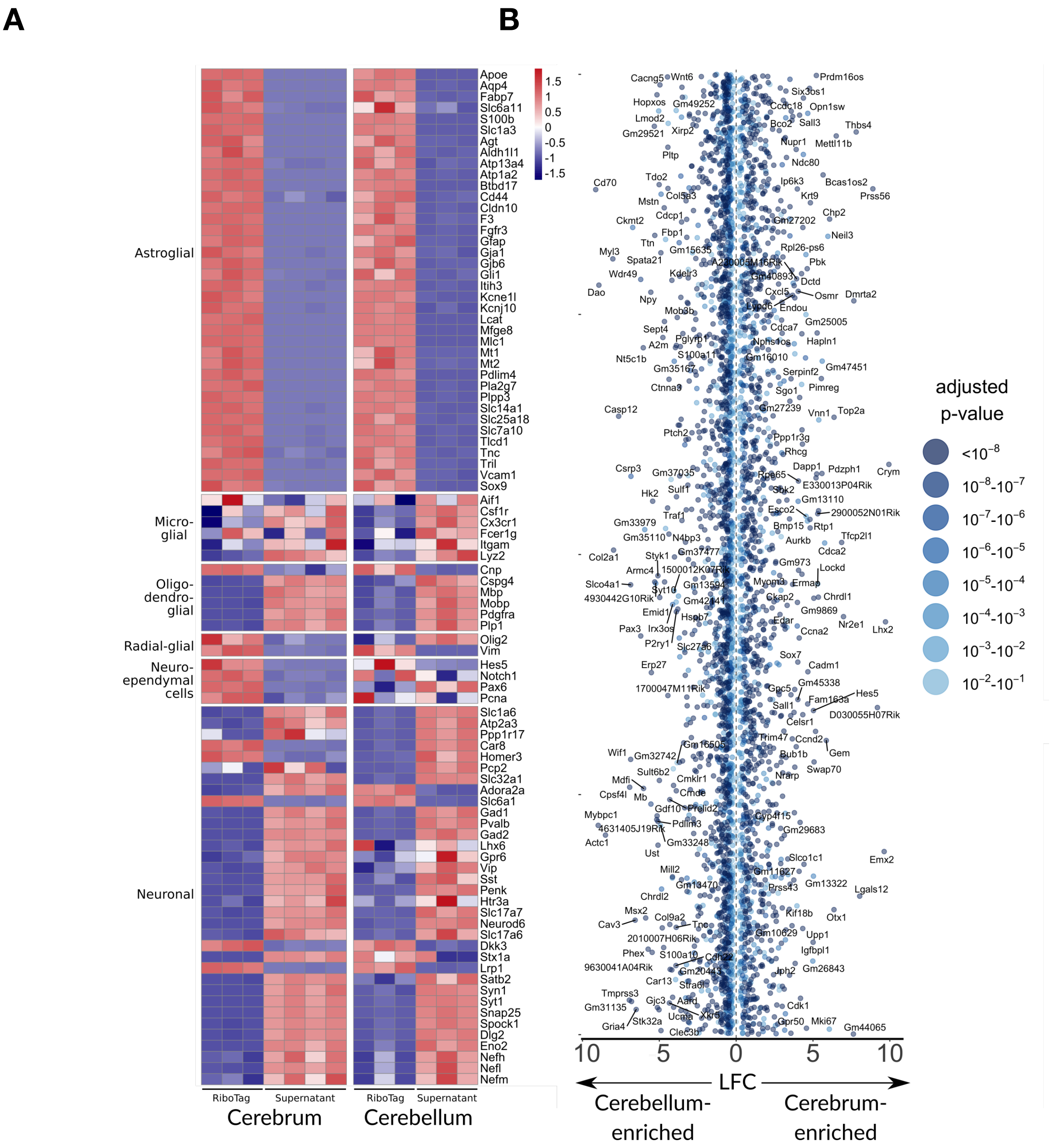

Supplementary figure 5. (A) Relative mRNA expression in cerebral and cerebellar astrocytes for a manually curated list of cell type marker genes. Color scale represents Z-score. **(B)** Top 100 of the most enriched genes in cerebellar (left side of the middle axis) and cerebral (right side of the middle axis) astroglia. LFC differences were calculated by comparing RiboTag IPs from the cerebrum with those from the cerebellum (or vice versa).

Table S1. PCR oligos for genotyping and verification of gene targeting.

ID (as in Fig 2B)	Name	Sequence	Binding to	Orientati on
P1	Cre_cDNA_for	GCATTACCGGTCGATGCAACGAGTG	CreERT2	F
P2	Cre_cDNA_rev	GAACGCTAGAGCCTGTTTTGCACGTTC	CreERT2	R
P3	Slc1a3_genomic_for	CAGCTCCTCCTGTATCCAGTGTTCT	Intron 9	F
P4	Slc1a3_genomic_rev	TACTCCCCCGCAGCCTAGTGTTA	Exon 10	R
P5	Slc1a3_rev	GAACAGTTTCCAACACTTGGTGCT	Exon 10	R
P6	Cre_for	GACACTTTGATCCACCTGATGGC	CreERT2	F
P7	Slc1a3_for	GGTCCTCACTGTTCACCTTCTGT	Intron 9	F
